# Interactions between tetrathiafulvalene units in dimeric structures – the influence of cyclic cores

**DOI:** 10.3762/bjoc.11.104

**Published:** 2015-06-02

**Authors:** Huixin Jiang, Virginia Mazzanti, Christian R Parker, Søren Lindbæk Broman, Jens Heide Wallberg, Karol Lušpai, Adam Brincko, Henrik G Kjaergaard, Anders Kadziola, Peter Rapta, Ole Hammerich, Mogens Brøndsted Nielsen

**Affiliations:** 1 Department of Chemistry, University of Copenhagen, Universitetsparken 5, DK-2100 Copenhagen Ø, Denmark; 2 Sino-Danish Centre for Education and Research (SDC), Niels Jensens Vej 2, DK-8000 Aarhus C, Denmark,; 3 Institute of Physical Chemistry and Chemical Physics, Faculty of Chemical and Food Technology, Slovak University of Technology, Radlinskeho 9, 81237 Bratislava, Slovak Republic

**Keywords:** alkynes, mixed valence, radiaannulene, tetraethynylethene, tetrathiafulvalene

## Abstract

A selection of cyclic and acyclic acetylenic scaffolds bearing two tetrathiafulvalene (TTF) units was prepared by different metal-catalyzed coupling reactions. The bridge separating the two TTF units was systematically changed from linearly conjugated ethyne, butadiyne and tetraethynylethene (*trans-*substituted) units to a cross-conjugated tetraethynylethene unit, placed in either acyclic or cyclic arrangements. The cyclic structures correspond to so-called radiaannulenes having both endo- and exocyclic double bonds. Interactions between two redox-active TTF units in these molecules were investigated by cyclic voltammetry, UV–vis–NIR and EPR absorption spectroscopical methods of the electrochemically generated oxidized species. The electron-accepting properties of the acetylenic cores were also investigated electrochemically.

## Introduction

Linking together two redox-active tetrathiafulvalene (TTF) units by a π-conjugated bridge has found immense interest in materials science, in particular in the quest for organic conductors [1–3]. Thus, the materials properties rely on the degree of intra- and intermolecular electronic interactions between neutral and oxidized TTFs. TTF-dimers can potentially exist in five redox states, 0, +1, +2, +3, and +4, depending on the degree of interactions between the two units. These five redox states have for example been observed in compounds with two TTF units fused to a central benzene or pyrazine ring [4]. If the two TTFs are oxidized at the same potentials in an electrochemical experiment, they behave instead as electrochemically independent redox centres with only weak Coulombic interactions between the cations. Interactions can also be tracked spectroscopically, and while two TTFs might be oxidized at the same or close potentials, the bridge may still convey communication between the units. Works by Otsubo, Iyoda and co-workers [5–7] have shown that two TTFs linked by ethynediyl and buta-1,3-diyndiyl behaved as electrochemically independent redox centres. However, the electronic spectra of the chemically generated radical cations (TTF-bridge-TTF^•+^) were reported to show clear intramolecular intervalence charge-transfer (IVCT) absorption bands with broad maxima around 1300–1400 nm [6].

**Figure 1:**
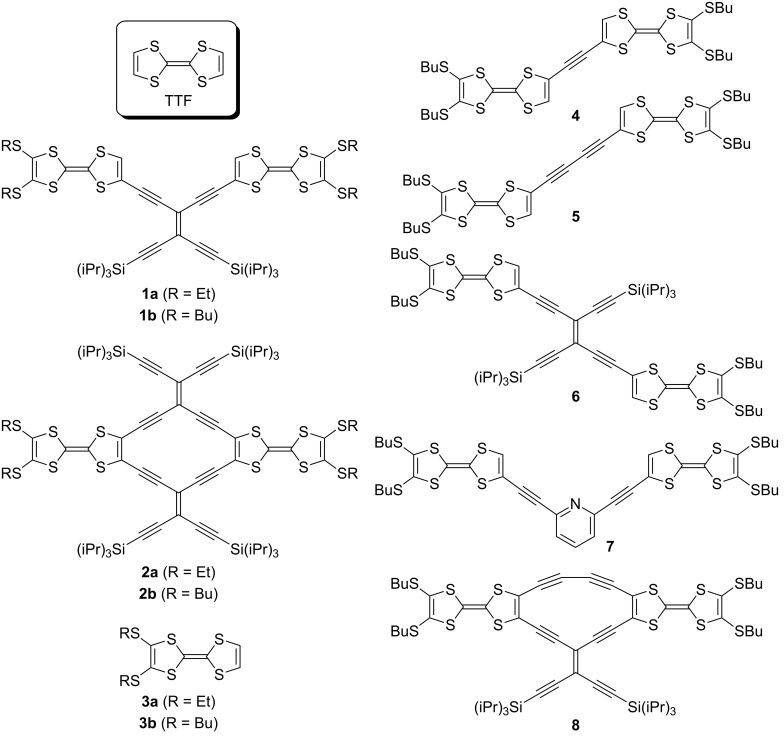
TTF dimers with linearly or cross-conjugated bridging units, acyclic or cyclic bridging units.

We have previously found that when separating two TTFs by a cross-conjugated tetraethynylethene (TEE) spacer as in compound **1a** (Figure 1), then the two TTFs behave as independent redox centres that are oxidized at the same potentials in cyclic voltammetry (two two-electron oxidations) [8–10]. However, by bridging the two TTFs with an additional TEE in a cyclic structure, as in the radiaannulene **2a**, communication between the two TTFs is observed in the cyclic voltammetry experiment, and the first two-electron event showed the waves diverging from each other (two stepwise oxidations) [10]. In addition, the intermediate radical cation showed an IVCT absorption at low energy (2257 nm). We became interested in elucidating the dependence on acyclic versus cyclic bridging units in more detail by cyclic voltammetry and EPR/UV–vis–NIR spectroelectrochemistry. With regard to conformational flexibility and the lack of it, it deserves mentioning that Low and co-workers [11–12] have shown how rotamers can indeed influence the electronic coupling in bis-ruthenium complexes separated by oligoynediyl spacers. In addition, an increased interaction between redox centres upon linking them together in cyclic structures was previously observed in ferrocene-dimers [13].

Here we present the synthesis and properties of a selection of TTF-dimers as shown in Figure 1. The known TTF **3b** [14] with butylthio substituents, ascertaining good solubility, was used as building block for these dimers. Although ethynediyl- and buta-1,3-diynediyl-bridged dimers were previously studied [1,5–7], we included **4** and **5** in the series in order to be able to compare properties measured under identical conditions. The two TEE-bridged dimers **1b** [15] and **6** have the TTFs arranged in either cross-conjugated or linearly conjugated arrangements. The pyridine-2,6-diyl-bridge present in compound **7** also connects two TTFs in a cross-conjugated pathway. The synthesis of radiaannulene **2b** was very recently described [15], and the properties of **2a** and **2b** are included here for comparison to those of the new unsymmetrical radiaannulene **8** that has a cross-conjugated TEE-diyl and a linearly conjugated buta-1,3-diynediyl bridge between the two TTFs.

## Results and Discussion

### Synthesis

**Scheme 1:**
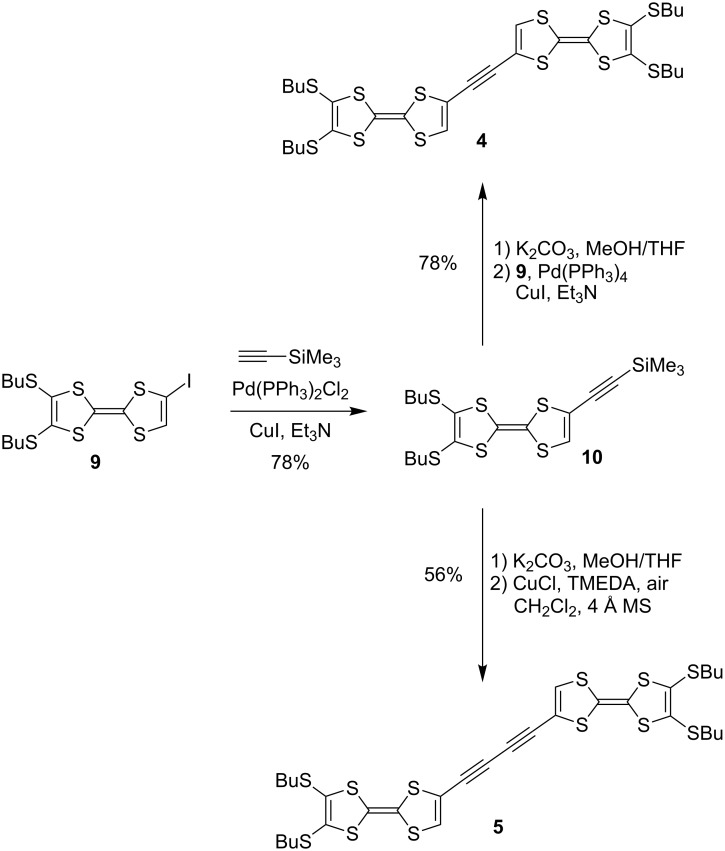
Synthesis of TTF dimers with alkyne bridges. TMEDA = *N*,*N*,*N’*,*N’*-tetramethylethylenediamine. MS = molecular sieves.

The synthesis of **4** and **5** was accomplished according to Scheme 1 starting from the known TTF-iodide **9**, readily prepared from compound **3b** [16]. A Sonogashira coupling with trimethylsilylacetylene gave **10** that after desilylation was either subjected to a Sonogashira coupling with **9** to give **4** or to an oxidative Hay coupling to give **5** – using recently developed conditions where 4 Å molecular sieves were added to the reaction mixture to remove water [17].

**Scheme 2:**
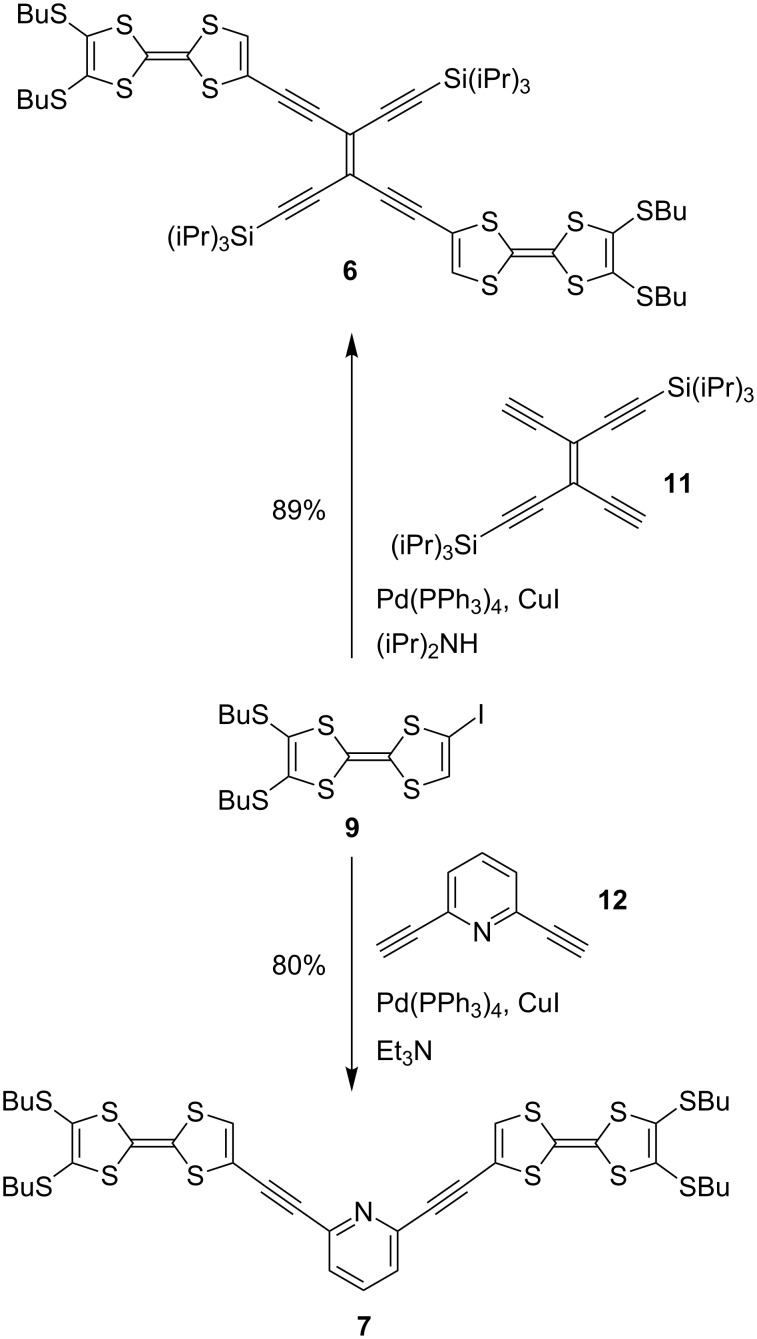
Synthesis of TTF dimers with TEE and diethynylpyridine bridges.

**Scheme 3:**
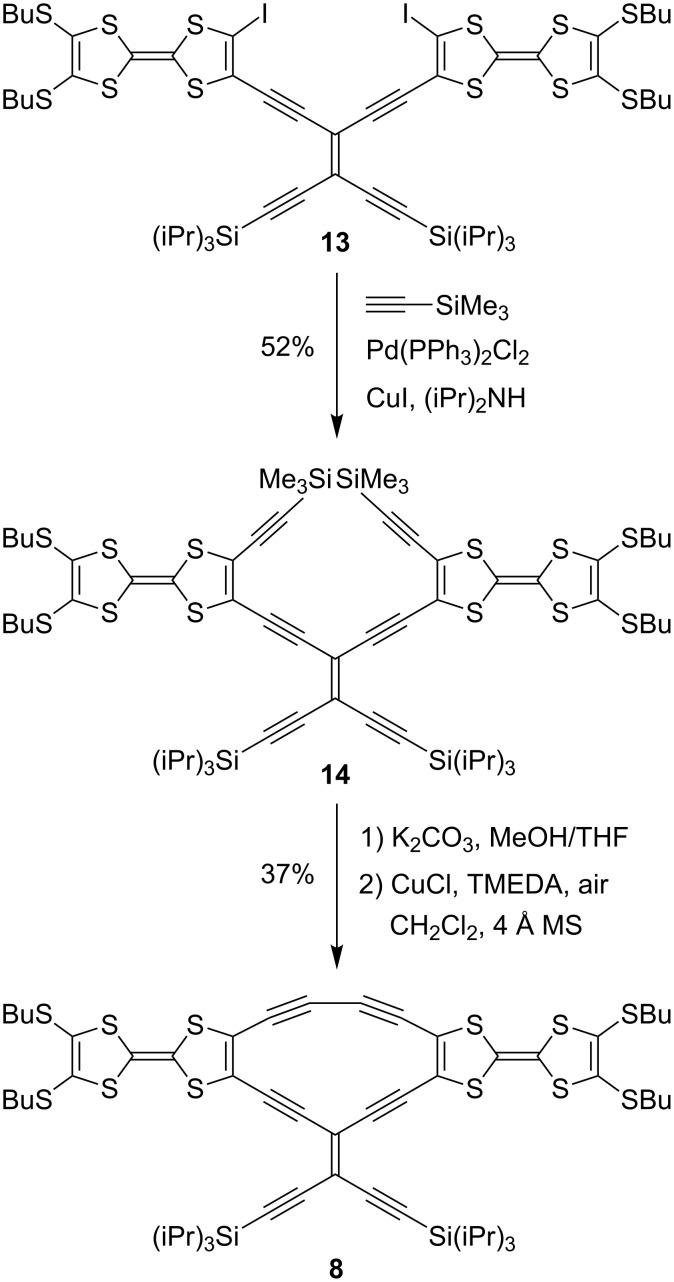
Synthesis of TTF dimer with radiaannulene core.

**Figure 2:**
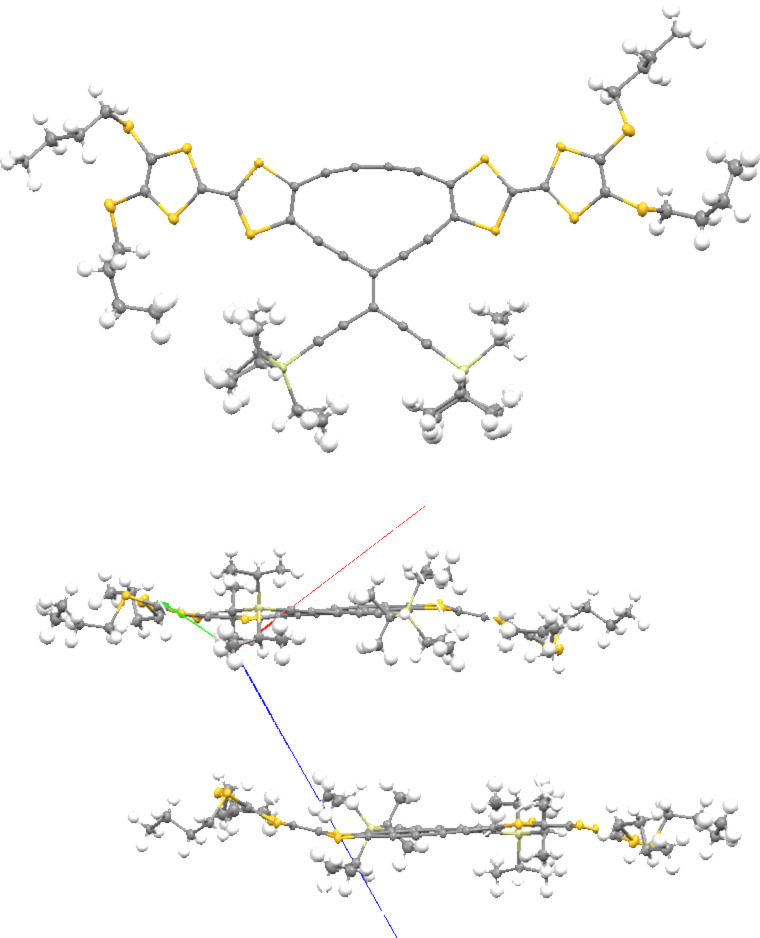
Molecular structure of **8** (top) and packing diagram (bottom). Crystals were grown from CH_2_Cl_2_/MeOH. CCDC 1050621 contains the supplementary crystallographic data.

**Figure 3:**
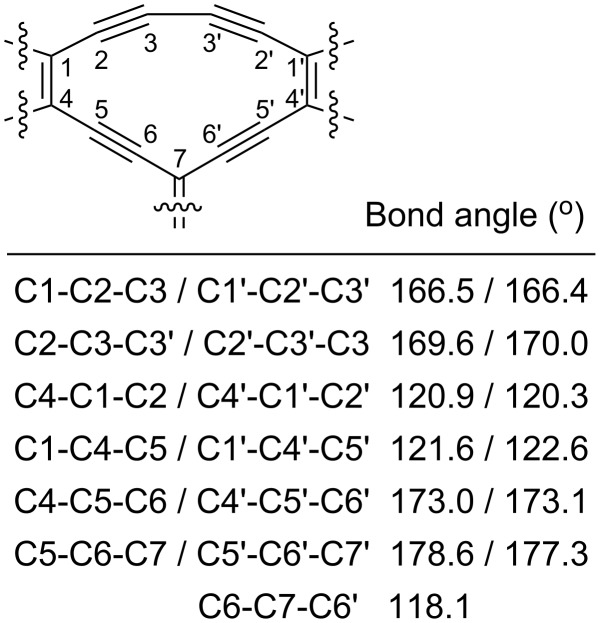
Bond angles for the cyclic core of **8** (X-ray crystal structure data).

Compounds **6** and **7** were prepared by treating **9** with either the *trans*-TEE **11** [18] or 2,6-diethynylpyridine **12** in Sonogashira coupling reactions (Scheme 2). The unsymmetrical radiaannulene **8** was prepared according to Scheme 3. The TEE-TTF derivative **13** [15] was reacted in a Sonogashira coupling with an excess of trimethylsilylacetylene to furnish the product **14**. Removal of the silyl groups and subjecting the intermediate to oxidative Hay conditions in the presence of 4 Å molecular sieves then gave the macrocyclic product **8** in good yield. The structure of **8** was confirmed by X-ray crystallographic analysis (Figure 2). The bond angles in the cyclic core are listed in Figure 3. The butadiyne-diyl unit is bent from linearity – with a C_TTF_–C≡C angle of 166° and a C≡C–C angle of 170°. The cyclic core is almost planar, while the two TTF units are slightly bent.

Compounds **1b**, **6**, and **8** containing TEE bridging units exhibit longest-wavelength absorption maxima at λ_max_ 529 nm (ε 5800 M^−1^cm^−1^), 554 nm (ε 12000 M^−1^cm^−1^), and 651 nm (ε 3900 M^−1^cm^−1^), respectively, in CH_2_Cl_2_ (for spectral data of all compounds, see Supporting Information File 1). For radiaannulene **2a** we previously found a longest-wavelength absorption of 644 nm (ε 11000 M^−1^cm^−1^) [10]. We assign these low-energy bands to charge-transfer absorptions, which are usually observed in π-conjugated systems containing TTF donors and large acetylenic scaffolds that behave as electron acceptors [8–10]. The significant redshifts of the charge-transfer absorptions experienced for **8** and **2a** relative to the absorptions of **1b** and **6** signal the stronger acceptor strength of radiaannulene cores (lower-lying LUMOs) relative to an acyclic TEE unit. This effect was also confirmed by electrochemistry (vide infra). Interestingly, compound **7** containing a diethynylpyridine acceptor unit exhibits the most redshifted absorption of all the compounds at λ_max_ 779 nm (ε 210 M^−1^cm^−1^), but of significantly lower intensity than the charge-transfer absorptions of the TEE compounds.

### Electrochemistry

The redox properties of the TTF-bridge-TTF molecules were investigated by cyclic voltammetry and differential pulse voltammetry in CH_2_Cl_2_ (0.1 M Bu_4_NPF_6_). For clarity we have broken the discussion of the electrochemistry into two sections, the first being the oxidation of the TTFs and their electronic interactions in comparison to related examples in the literature. The other section deals with the reduction of the molecules.

**Figure 4:**
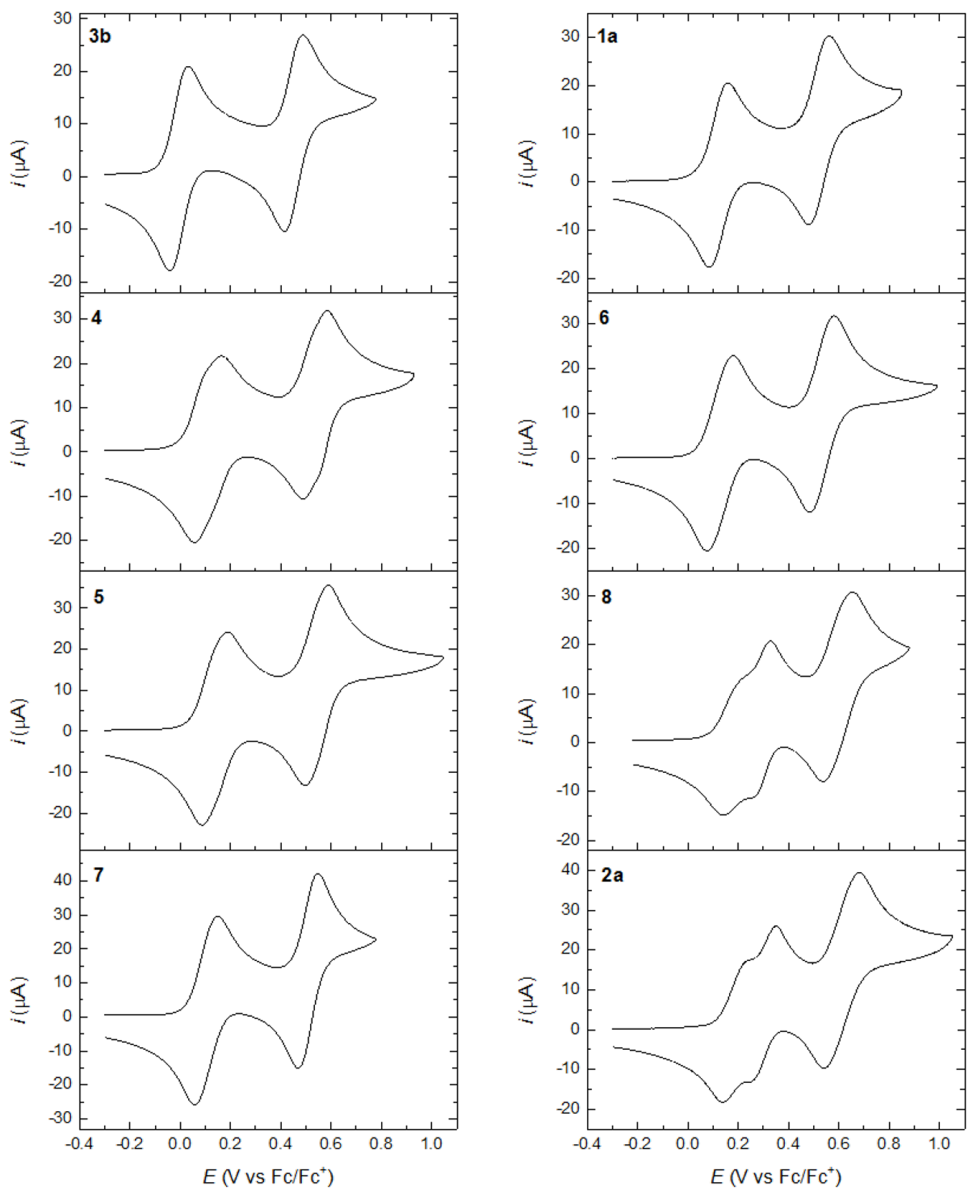
Cyclic voltammograms obtained for the oxidation of compounds **1a** ([10]), **2a** ([10]), and **3b**–**8** (this work, ca. 1 mM) in CH_2_Cl_2_ (0.1 M Bu_4_NPF_6_) at a glassy carbon electrode with a scan rate of 0.1 V s^−1^.

**Oxidation:** Cyclic voltammograms of compounds **1a**, **2a**, **3b**, and **4–8** measured at a glassy carbon electrode are shown in Figure 4 [19] and redox potentials are listed in Table 1 together with the shortest TTF (C=C)–TTF (C=C) distance in each structure (obtained from the crystal structures or from DFT calculations). In addition to half-wave potentials obtained from cyclic voltammetry, the table includes peak potentials from differential pulse voltammetry (see Supporting Information File 1, Figure S3). The half-wave redox potentials have been additionally determined for samples **1b** and **2b** (Table 1 and Supporting Information File 1, Figure S4) using cyclic voltammetry (with a platinum-wire working electrode in the presence of internal ferrocene potential marker). They are very close to **1a** and **2a**, respectively, as expected. Using **3b** as a reference compound, it is seen that all the other compounds are slightly more difficult to oxidize owing to the presence of the electron-withdrawing alkyne system. The acyclic compounds **1a** and **5**–**7** all undergo two reversible two-electron oxidations; that is, the two TTFs are oxidized at the same potentials when separated by butadiyne, *gem*- or *trans*-TEE, or bis-ethynylpyridine linkers. When two TTFs are closer together via an ethyne spacer (**4**), the two waves are rather broad. In fact, a shoulder can be seen for each wave, which indicates weak interactions between the two units, not only for generating two TTF radical cation units, but also for the final generation of two TTF dication units. This splitting is seen more clearly in the differential pulse voltammograms (Supporting Information File 1, Figure S3). We note that for the SEt derivative of compound **4**, measured under different conditions by Iyoda and co-workers (in PhCN, Bu_4_NClO_4_), only two two-electron oxidations were reported [6]. For the radiaannulene **2a** removal of two electrons was previously [10] found to occur stepwise with a separation between the first and second oxidations of 90 mV. Thus, despite the long *gem-*TEE bridging units, there is a weak interaction between the TTFs that are separated by a shortest distance of 6.64 Å according to the X-ray crystal structure [10]. For the other radiaannulene, **8**, the first oxidation of each TTF unit was also split into two waves, again indicating interaction between them. In this molecule, the two TTF units are separated by a distance of 6.31 Å.

**Table 1:** Electrochemical data for the oxidation of compounds **1**–**8**.^a^

	Cyclic Voltammetry (CV)			Differential Pulse Voltammetry (DPV)			Shortest TTF–TTF distance / Å
	
	*E*_ox_^1^ / V	*E*_ox_^2^ / V	*E*_ox_^3^ / V	*E*_p_^1^ / V	*E*_p_^2^ / V	*E*_p_^3^ / V	

**1a**^b^	+0.12 (2e)	+0.52 (2e)		+0.11 (2e)	+0.51 (2e)		6.88^c^
**1b**^d^	+0.10 (2e)	+0.50 (2e)					
**2a**^b^	+0.20 (1e)	+0.29 (1e)	+0.61 (2e)	+0.18 (1e)	+0.29 (1e)	+0.61 (2e)	6.64^e^
**2b**^d^	+0.18 (1e)	+0.25 (1e)	+0.57 (2e)				
**3a**^b^	−0.01 (1e)	+0.44 (1e)					n/a
**3b**	+0.03 (1e)	+0.48 (1e)		−0.01 (1e)	+0.44 (1e)		n/a
**4**	+0.11^f^ (2e)	+0.54^f^ (2e)		+0.08 / +0.12^f^	+0.54^f^/+0.56		4.04^c^
**5**	+0.14 (2e)	+0.54 (2e)		+0.13 (2e)	+0.55 (2e)		6.63^c^
**6**	+0.13 (2e)	+0.53 (2e)		+0.13 (2e)	+0.50 (2e)		8.92^c^
**7**	+0.15 (2e)	+0.55 (2e)		+0.10 (2e)	+0.51 (2e)		9.27^c^
**8**	+0.19^g^ (1e)	+0.27^g^ (1e)	+0.57 (2e)	+0.21 (1e)	+0.26 (1e)	+0.58 (2e)	6.31^e^

^a^Recorded at a glassy carbon working electrode (if not otherwise stated) in CH_2_Cl_2_ (0.1 M Bu_4_NPF_6_), potentials are given vs Fc/Fc^+^. CV scan rate: 0.1 V s^−1^. DPV step potential: 0.0045 V. DPV modulation amplitude: 0.025 V. ^b^Data from [10]. ^c^Distance(s) obtained by DFT calculations at B3LYP/cc-pVDZ level of theory. ^d^Recorded at platinum-wire working electrode. ^e^Distance obtained from X-ray crystal structure. ^f^Broad signal. ^g^Shoulder.

**Figure 5:**
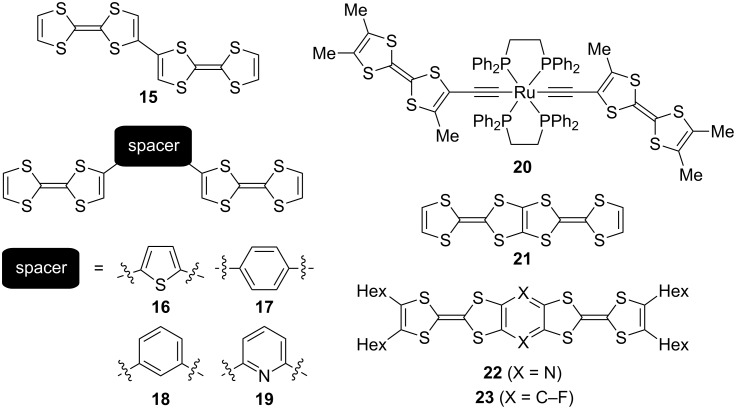
Selected bis-TTFs from literature [20–23].

Several bis-TTF molecules and their electrochemical properties have previously been reviewed [1–3]. Here we make a brief discussion of some selected structures of relevance to those studied in this work, as shown in Figure 5. For the directly linked compound **15** [20] and the aryl/heteroaryl mono-bridged compounds **16**, **17** [20] and **18**, **19** [21], only two two-electron oxidations were observed (in PhCN, Bu_4_NClO_4_). However, incorporation of a Ru centre between two TTF acetylides, complex **20** [22], has been shown to result in a splitting of both the first and second two-electron oxidations of the TTF units (by 110 mV for both). A fifth one-electron event associated to the Ru metal centre was also observed. The two TTFs are 9.37 Å apart, hence further than in the related alkyne compounds **4** and **5**. The strong communication between TTF units in **20** is mediated by the Ru centre, and the bulky ligands possibly also lock the molecule into a conformation where the two TTF units are in co-planarity [22]. The communication dramatically increases when the two TTFs are fused together as in compound **21** [23] or to either a pyrazine or benzene ring [4]. Thus, compounds **21**–**23** all show four distinguishable one-electron oxidation events; in our series, this was only to some extent seen for the C_2_-bridged compound **4** as the cyclic radiaannulene cores present in **2a** and **8** only promoted splitting of the first oxidation wave.

When taking all the results together, there seem to be some important trends. Placing two TTF units close together by a single bridge does not by itself seem to increase significantly the electronic communication when stepwise oxidations are used as a measure hereof. Instead, fusing directly together the two TTF units, connecting them to a benzene or pyrazine ring, or even to a larger radiaannulene ring, renders them independent redox centres, in all cases when it comes to the first one-electron oxidation of each TTF. Co-planarity of the TTF units seems to play a key role in these structures. This can also be enforced by sterical means and may explain the behaviour of **20**; thus, here the bulky 1,2-bis(diphenylphosphino)ethane (dppe) ligands and the Me group on the TFF might restrict the rotation around the triple bond and sterically keep the two TTFs in the same plane as revealed by the X-ray crystal structure [22]. In compound **18**, the two TTFs are only separated by a *meta*-phenylene bridge, but according to X-ray crystallography they are not co-planar, and no electronic interactions were observed [21]; the cross-conjugated nature of the bridge needs of course to be taken into account here as well. For the derivative of **4** with SEt end-groups instead of SBu groups, we calculate a barrier for rotating the two TTF units around the ethyne bridge of 2.5 kJ mol^−1^ (see Supporting Information File 1). The electronic interactions between two TTFs being spanned by alkynyl or poly-alkynyl bridges are quite poor in comparison to other redox groups. Ferrocene units are only marginally better than TTFs, but the interaction can here also be increased by “doubly-bridging” them [13]. Those redox groups that have shown the best interaction along the all-carbon bridges are the organometallic metal centres Re(NO)(PPh_3_)Cp* [24], Ru(dppe)Cp* [25], and Fe(dppe)Cp* [26] (Cp* = 1,2,3,4,5- pentamethylcyclopentadienyl), showing good communication still well past five carbon–carbon triple bonds between the metal centres.

**Figure 6:**
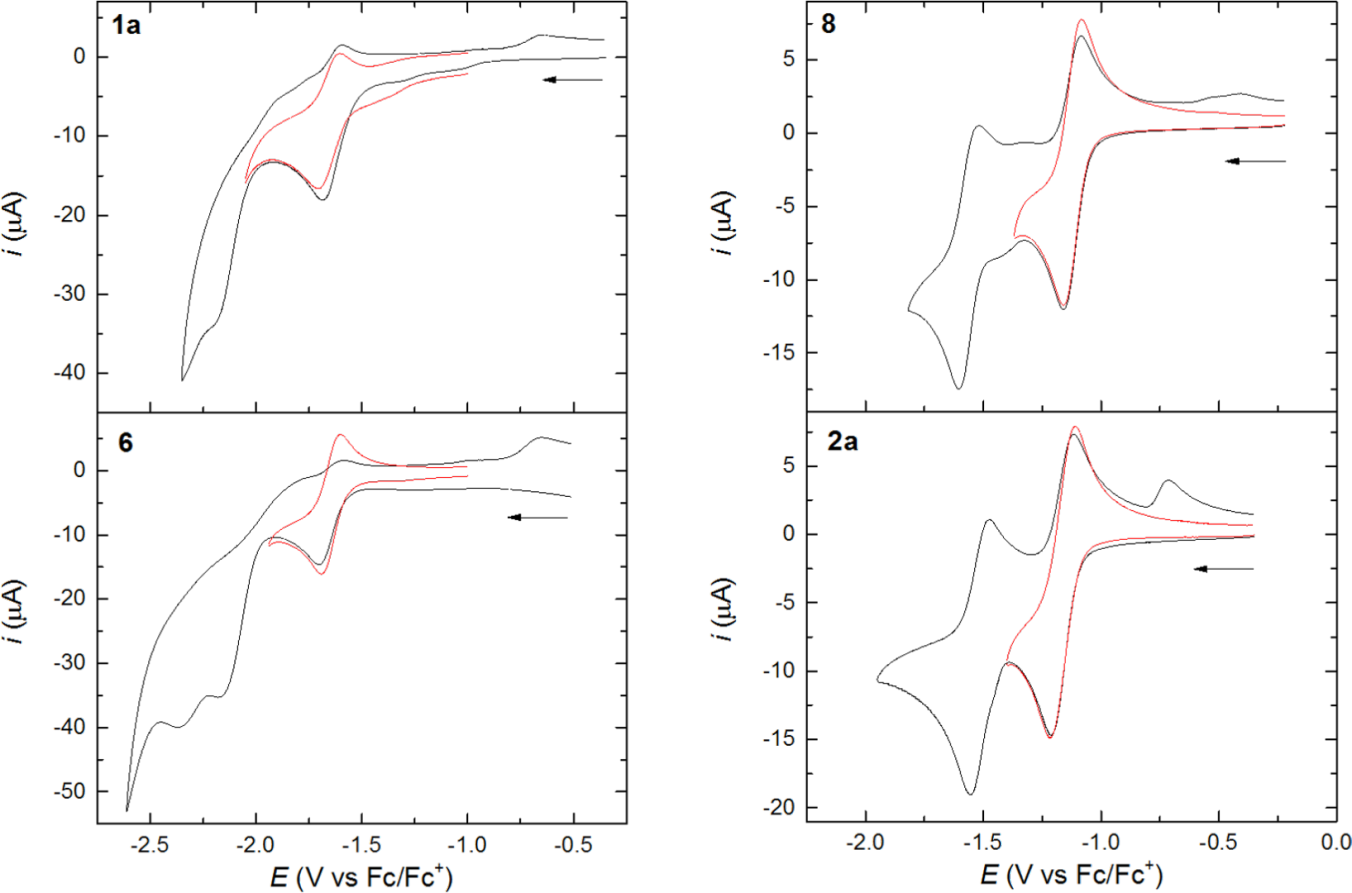
Cyclic voltammograms obtained for the reduction of compounds **1a**, **2a**, **6**, and **8** in CH_2_Cl_2_ (0.1 M Bu_4_NPF_6_) at a glassy carbon electrode with a scan rate of 0.1 V s^−1^. The reduction to the radical anion state only is shown in red color.

**Figure 7:**
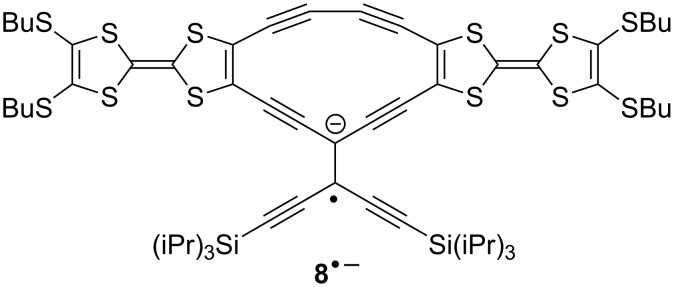
One possible resonance form of the radical anion of **8** with a 14 π_z_ aromatic core.

**Reduction:** As expected, compound **3b** was found to be non-reducible within the potential window defined by the solvent-supporting electrolyte system (CH_2_Cl_2_, Bu_4_NPF_6_). The reduction of the remaining seven compounds falls into three groups; the potentials have been summarized in Supporting Information File 1, Table S2. Compounds **4**, **5**, and **7**, without TEE cores, were found difficult to reduce with poorly defined reduction peaks being observed in the region −2.3 to −2.5 V (Supporting Information File 1, Figure S6). Current corresponding to the oxidation of the initially formed radical anions was not observed during the reverse scan for any of these three compounds. Minor oxidation peaks around −0.75 V may be attributed to the oxidation of intermediate anions presumably formed by further reduction of radical species resulting from protonation by residual water of the radical anions or by protonation of dianions [27]. The corresponding differential pulse voltammograms are shown in Supporting Information File 1, Figure S6. Figure 6 shows the cyclic voltammograms of the four compounds **1a**, **6**, **8** and **2a**, for which reverse current corresponding to the oxidation of the primarily formed radical anions is clearly seen (DPVs are found in Supporting Information File 1, Figure S5). The further reduction to reactive dianions is seen for **1a** and **6** and, as before, small oxidation peaks, presumably of the same origin as those observed for **4**, **5**, and **7** are seen around −0.75 V during the reverse scan. Most striking is the behaviour of **8** and **2a** for which reversible reduction, not only to the radical anions, but also to the dianions, is clearly seen although even in these two cases the dianions are sufficiently basic to produce minor amounts of the monoprotonation products, the oxidation of which are seen during the reverse scan. We have previously shown that the radiaannulene **2a** is reduced in two sequential one-electron steps at potentials significantly less negative, −1.16 V and −1.52 V vs Fc/Fc^+^, than that required to reduce the acyclic TEE compound **1a** (−1.70 V vs Fc/Fc^+^) [10]. We explained this readiness of **2a** to accommodate electrons by a gain of aromaticity; a resonance form with a 14 π_z_ core can be drawn for the dianion and a diatropic ring-current was supported by NICS calculations. The new radiaannulene **8** underwent reversible reductions at −1.12 V and −1.51 V. The readiness of the first reduction may again be explained by the generation of a 14 π_z_ aromatic core of the radical anion as shown in Figure 7. Thus, again the first reduction occurred at much less negative potential than that of its acyclic components, **1a** and **5** (−1.65 V and −2.35 V vs Fc/Fc^+^).

### UV–vis–NIR and EPR spectra of oxidized species

**Scheme 4:**
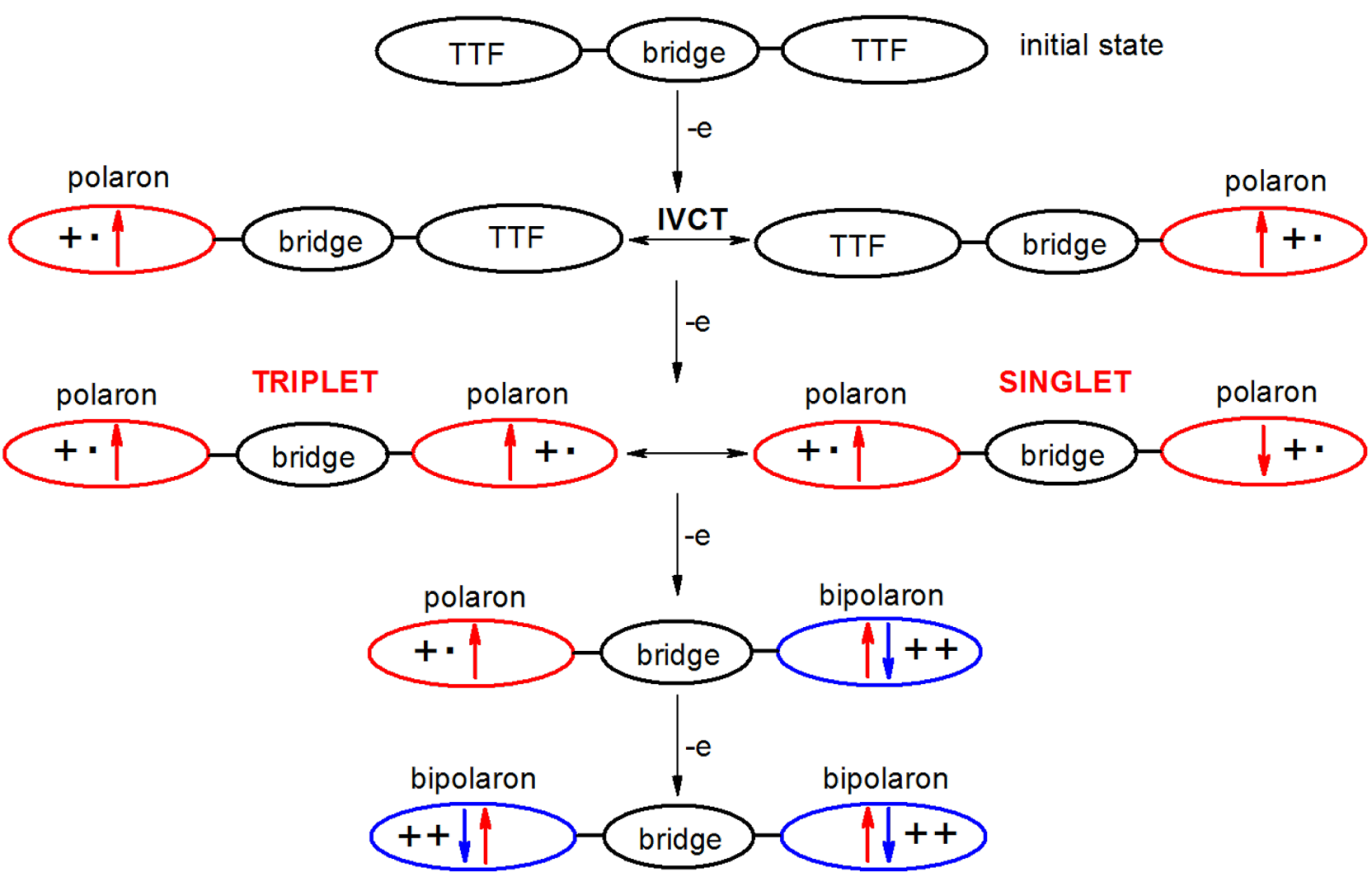
Spin–spin interactions resulting from oxidation of TTFdimers.

The EPR and optical (UV–vis–NIR) properties of the oxidized species were studied by in situ spectroelectrochemistry in CH_2_Cl_2_ (Bu_4_NPF_6_) at a platinum mesh electrode. Even at low scan rate of 2 mV s^−1^ used for these studies, the reversibility remains unchanged confirming the high stability of the formed radical cation and dication states within each TTF redox site (in further text defined as “polaron” and “bipolaron” states, respectively; see Scheme 4). The small peak separation, Δ*E*, between the neighbouring voltammetric peaks for the first and the second electron transfer as well as for the third and the fourth electron transfer indicates a weak (but still certain) interaction between the two TTF units in the investigated TTF dimers.

**Figure 8:**
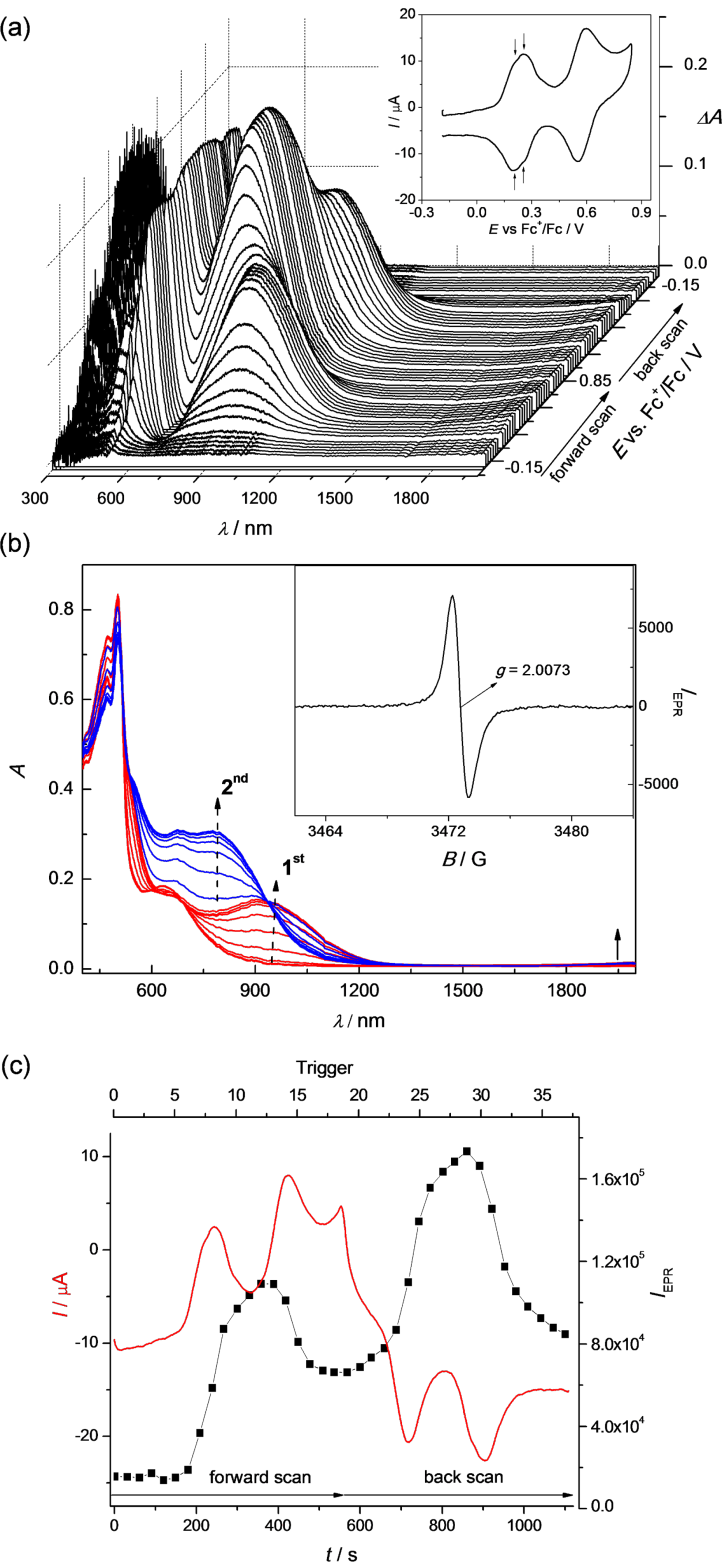
In situ EPR−UV–vis–NIR cyclic voltammetry of **2b** (1 mM) (a) potential dependence of difference vis–NIR spectra with the corresponding cyclic voltammogram (in CH_2_Cl_2_/0.1 M Bu_4_PF_6_, scan rate v = 2 mV s^−1^; the arrows indicate separation between the first and second oxidations); (b) evolution of vis–NIR spectra in forward scan in 2D projection in the region of the first (red lines) and the second (blue lines) voltammetric double peak (inset: representative EPR spectrum of radical species monitored upon oxidation); (c) time dependence of the current (red line) and double-integrated EPR intensity (black solid squares).

**Figure 9:**
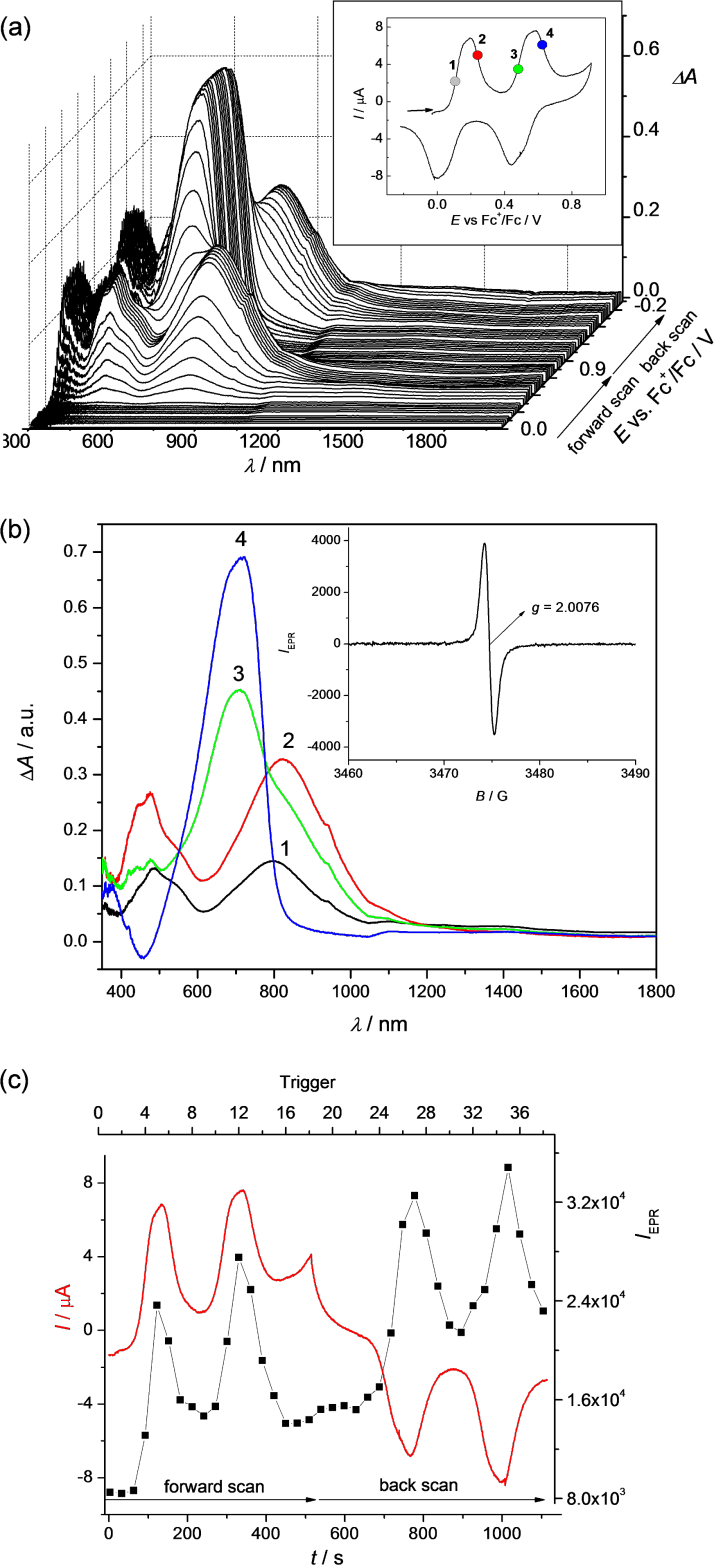
In situ EPR−UV–vis–NIR cyclic voltammetry of **4** (0.4 mM): (a) potential dependence of difference vis–NIR spectra with corresponding cyclic voltammogram (in CH_2_Cl_2_/0.1 M Bu_4_PF_6_, scan rate v = 2 mV s^−1^); (b) selected vis–NIR spectra observed at the foot of the first anodic double peak (1), behind the first voltammetric double peak (2), at the foot of the second anodic double peak (3) and behind the second voltammetric double peak (4); see coloured marked circles in the corresponding voltammogram (inset: representative EPR spectrum of radical species monitored upon oxidation); (c) time dependence of the current (red line) and the double-integrated EPR intensity (black solid squares).

During the in situ oxidation of **1b**, **2b**, **4**, **5**, **6**, and **8** at the first voltammetric double peak, two dominating optical transitions arise, the first one in the region 700–950 nm and the second one in the region 450–550 nm, both characteristic of TTF cation radical (polaron) species [28–29], as illustrated for compound **2b** in Figure 8 and for compound **4** in Figure 9. Simultaneously, singlet EPR spectra with *g*-values around 2.007 were observed (see insets of Figures 8b and 9b) confirming delocalized spin predominantly within the TTF moieties. These data confirm that in the region of the first voltammetric double peak, the only polaronic states are formed in the TTF-dimer. At the second voltammetric double peak, a new dominating absorption band in the region 600–900 arises for all investigated dimers, which corresponds to the formation of doubly charged (bipolaronic) TTF moieties.

**Figure 10:**
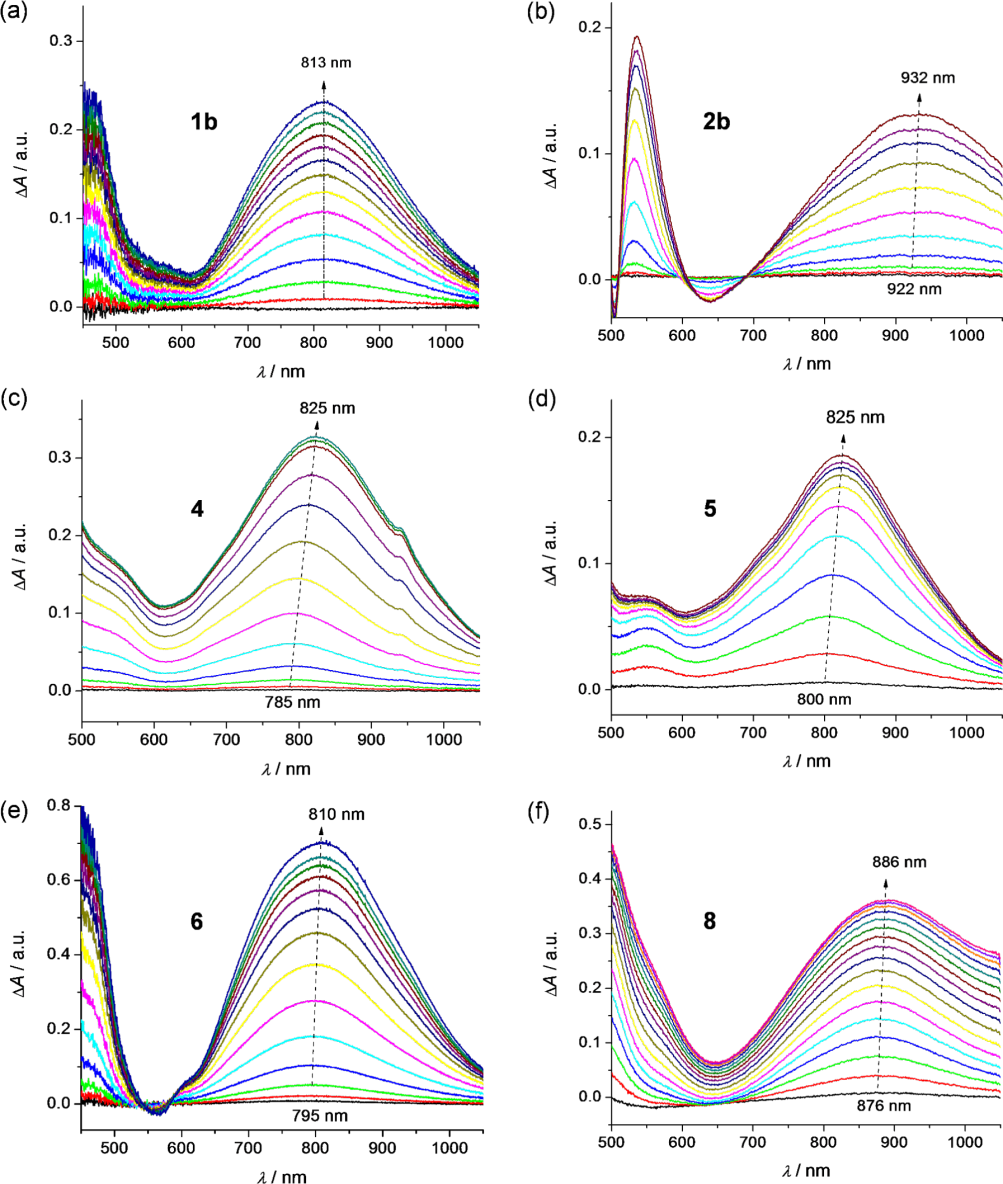
Vis–NIR spectral changes observed during anodic oxidation of each TTF unit to cation radical within the first voltammetric double peak: TTF–TTF → TTF^•+^–TTF → TTF^•+^–TTF^•+^.

Interestingly, it seems that by increasing the interaction between the polaronic species in the dimer we can observe a slight shift of the optical transition characteristic of TTF radical cation going from the first to the second electron transfer as shown in Figure 10. The strongest shifts were observed for dimers **4** and **5** while no shift was found for dimer **1b**. These correspond to Davydov redshifts of head-to-tail TTF^•+^–TTF^•+^, signalling intramolecular interactions rather than face-to-face intermolecular interactions [2,30]. Additionally, although all investigated TTF dimers show quite analogous electrochemical and UV–vis–NIR spectroelectrochemical response, the EPR spectroelectrochemical behaviour differs to a certain extent (compare Figure 8c and 9c). Due to a very weak interaction between both mono-charged TTF units in **2b**, the optical spectra of both mono-charged and doubly-charged TTF dimers are very similar and no remarkable changes both in the optical and the EPR spectra were observed in the region of the first cyclovoltammetric double peak. The EPR intensity reaches its maximum behind the first voltammetric double peak (Figure 8c). The maximum of the low energy band of the polaronic TTF species in **2b** is only slightly shifted from 922 nm to 932 nm (small Davydov redshift) upon oxidation in the region of the first voltammetric double peak (Figure 10b). Going to the region of the second voltammetric double peak, the EPR intensity starts to decrease with the minimum behind the second voltammetric double peak, proving the EPR silent character of the TTF dimer tetracation with the optical band at λ_max_ = 810 nm (Figure 8b). Analogous EPR-spectroelectrochemical behavior was observed for compounds **1b**, **6**, and **8** (see Supporting Information File 1, Figures S7–S9). It should be noted that almost the same EPR spectroelectrochemical response was found for concentrated as well as for much diluted solutions (see Supporting Information File 1, Figure S10 for illustration, conc. of **8** at 1 mM vs 0.05 mM).

In EPR–UV–vis–NIR cyclovoltammetric experiments of compounds **4** and **5**, the potential dependencies of the EPR signal are different in comparison to compounds **1b, 2b**, **6**, and **8**. The EPR intensity reaches two maxima in both the forward and back scans. The minimum of EPR intensity was monitored at the second electron transfer as well as at the fourth electron transfer as illustrated for compound **4** in Figure 9c. The increase of the absorbance at dominating band around 790 nm found at the foot of the first anodic double peak for compound **4** corresponds to the increase of the EPR intensity. Simultaneously, a new optical band at 485 nm appears with an additional low-intense NIR band at ca. 1400 nm (see Figure 9b), confirming the primarily formed monocharged dimer **4**^•^**^+^**, which exhibits an IVCT band as also observed by chemical [6] or electrochemical oxidation (vide infra; also **2b**^•+^ exhibited an IVCT band, with maximum >2000 nm, as previously reported for the related species **2a**^•+^ [10]). By increasing the electrode potential to the value of the second electron transfer (still in the region of the first anodic double peak), a quite strong shift of the low energy transition from 785 nm to 825 nm (Figure 10c) was observed with simultaneous decrease of the corresponding EPR intensity. Changing the concentration from 0.4 mM to 0.05 mM for sample **4**, the same EPR spectroelectrochemical response was found (see Supporting Information File 1, Figure S11 for illustration). The analogous behaviour was found for compound **5** with the wavelength shift of the low optical band from 800 nm to 825 nm (see Figure 10d and Supporting Information File 1, Figure S12). The EPR silent character of TTF dimer dications found for samples **4** and **5** indicates a substantial interaction of the formed polaronic states leading to the EPR silent species. Interestingly, at the foot of the second anodic double peak, actually corresponding to the third electron transfer, an increase of EPR intensity was monitored confirming that trication of the TTF dimer is paramagnetic. It should be noted that at this anodic potential both optical transitions from polaronic and bipolaronic species are present (see green line 3 in Figure 9b). This confirms that one TTF unit is in its polaronic form while the second one is bipolaron (see Scheme 4). At the potentials behind the second voltammetric double peak, where the absorbance at 715 nm shows a maximum (see blue line 4 in Figure 9b), the EPR intensity has a minimum confirming the EPR silent character of bipolaronic TTF species and that TTF dimer tetracation is EPR silent as expected.

The complex spectroelectrochemical responses may be explained by two possible mechanisms. *The first one* concerns an intramolecular interaction of two polaronic TTF units leading to the EPR silent polaron pair in the singlet state. *The second possibility* could be the formation of intermolecular π-dimers [28,31] represented by the EPR silent tetracationic structures formed from the two doubly charged TTF dimers. After the third electron transfer to each TTF dimer these structures are no more stable and the EPR active TTF dimer trications are formed, which are further immediately charged to the EPR silent TTF dimer tetracations.

As already extensively discussed in [32] concerning the redox properties of self-assembled polyelectrolyte multilayers, consisting of well-defined water-soluble electronically conducting poly-3-(30-thienyloxy)propyltriethylammonium, the spinless doubly charged species can be explained by the interchain or intrachain coupling of two polarons, leading to the formation of dimeric structures between the neighboring chains or to the formation of two polaronic structures in a singlet ground state within the chain [33]. The structures corresponding to polarons and polaron pairs exhibited similar optical spectra and only a small shift in wavelengths was found [32]. It should be noted that the formation of interchain π-dimers is strongly suppressed in dilute solutions. As already mentioned above, we observed the same potential dependence of the EPR signal for compounds **4** and **5** both in the concentrated and in the diluted solutions. This suggests that the doubly charged structures **4** and **5** are in the form of a spinless dication, represented by the two interacting intrachain polarons. Obviously, the optical spectra of interchain π-dimers of short oligomers are characterized by two optical transitions, which exhibit a rather large blue shift with respect to the two bands of the corresponding radical cations which was not observed for TTF-dimers **4** and **5**.

Going to the region of the second voltammetric double peak, when both TTF segments are already monocharged, the bipolaronic EPR silent TTF structures start to be formed and a new optical band appears in the spectral region between the two polaronic bands (see blue line 4 in Figure 9b and Scheme 4).

For compound **8**, in addition to the small shift of the TTF cation optical band from 876 nm to 886 nm (Figure 10f), we observed also a small shift of *g*-value going to the second electron transfer (see Supporting Information File 1, Figure S9d). This indicates the non-negligible interaction between the polaronic states within the doubly charged TTF dimer **8** but this interaction does not lead to the spinless species as found for **4** and **5**. Both polaronic structures behave as nearly independent and their weak interaction probably leads to the shift in the *g*-value.

**Figure 11:**
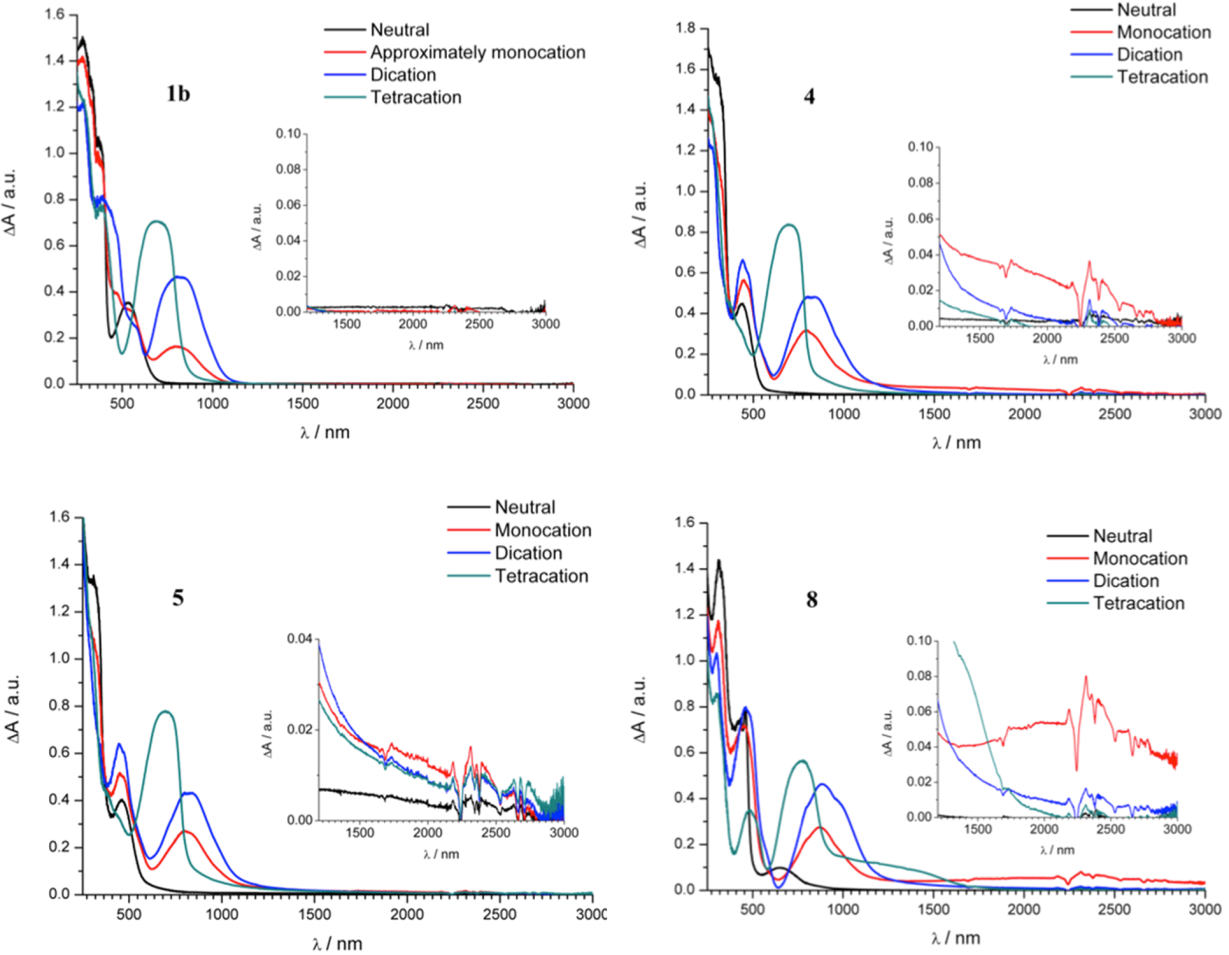
UV–vis–NIR absorptions of **1b** (2.4 mM), **4** (3.5 mM), **5** (2.9 mM), and **8** (1.9 mM) in CH_2_Cl_2_ + 0.1 M Bu_4_NPF_6_ at different oxidation states (obtained by electrolysis of the neutral species in an Ottle cell, ultimately generating the tetracation). The insets show expansions of the NIR region. For **1b**, the assignment “approximately monocation” refers to the stage during the electrolysis where this cation is judged to be the major species present.

**NIR absorptions of monocations.** Finally, we wanted to examine, in more detail, the broad bands in the NIR region of the spectra of the mixed-valence radical cations. For this objective, compounds **1b**, **4**, **5**, and **8** dissolved in CH_2_Cl_2_ + 0.1 M Bu_4_NPF_6_ were studied using another spectroelectrochemical set-up, an optically transparent thin-layer electrochemical Ottle cell equipped with a Pt mini grid working electrode and CaF_2_ windows. Figure 11 shows the spectral evolution upon electrolysis of **1b**, **4**, **5**, and **8**. Not surprisingly, compound **1b**, which showed no sign of electronic interactions between the TTF units in the CV (two two-electron oxidations), exhibited no CT absorption band during the oxidation from neutral to dication. Instead, compound **8**, and, in part, compound **4** showed interactions during the first oxidation of each TTF in the cyclic and differential pulse voltammetry experiments. In accordance hereto, both **4**^•+^ and **8**^•+^ exhibited broad absorption bands extending to 3000 nm (and apparently beyond for **8**^•+^, but, in general, baseline fluctuations add some uncertainty to the intensities of the NIR absorptions and where they exactly end in our spectra). The maximum is around 2300 nm for **8**^•+^ (the noise present is due to strong interference from vibrational transitions in the CH_2_Cl_2_ solvent), while it is somewhat blueshifted for **4**^•+^ and seems to agree with the value 1400 nm estimated from the parallel measurement described above (Figure 9b). The NIR absorption of **4**^•+^ is in agreement with the broad absorption at 1300 nm reported by Iyoda and co-workers [6] for a derivative of **4** with SEt substituents (measured at one order of magnitude lower concentration, <10^−4^ M, in MeCN/CH_2_Cl_2_ 1:4 using Fe(ClO_4_)_3_ as oxidizing agent). It is difficult to say if the long and weak absorption tail extending to almost 3000 nm observed in our experiment could be an IVCT absorption of the intermolecular mixed valence species, if present. The NIR absorptions of **4**^•+^ and **8**^•+^ disappeared upon further oxidation to the dications, corroborating the CT character. The position of the more well-defined NIR absorption band of **8**^•+^ is close to that of **2a**^•+^ at 2257 nm [10]; **2b**^•+^ also experiences this absorption according to the EPR-spectroelectrochemistry described above (see Figure 8), but in that experiment, the NIR detection only goes to 2000 nm and therefore does not provide the full NIR absorption band. The neutral spectrum of **8** was not fully regenerated after one cycle (neutral – monocation – dication – tetracation – neutral), presumably due to some degradation (see Supporting Information File 1) although an air-tight Ottle cell was used, filled with solution kept under inert atmosphere. When a TEE is separating the two TTFs in an acyclic structure (**1b**^•+^), they are too remote to interact. However, when cyclized into TEE-based radiaannulene structures, **2a**^•+^, **2b**^•+^, and **8**^•+^, interactions appear despite the similar distance between the TTF units as in **1b**. The triisopropylsilyl groups in **2a** and **2b** prevent the formation of face-to-face complexes in which the TTF units interact pairwise (calculations indicate that the TTF units cannot come closer together than approximately 13 Å, see Supporting Information File 1) and the NIR absorptions of the radiaannulene cations are therefore most likely of intramolecular origin. No interaction between the two TTF units was observed in the CV of **5**. There seems, however, to be a very weak CT band around 1700 nm for **5**^•+^ with a tail until ca. 3000 nm (but this weak absorption does not disappear fully upon further oxidation). In comparison, Iyoda and co-workers [6] reported a broad absorption at 1200 nm for the monocation of the SEt derivative obtained by chemical oxidation. We also subjected selected compounds to chemical oxidations using tris(4-bromophenyl)aminium hexachloridoantimonate (“magic blue”) as oxidant (spectra not shown), but weak NIR absorption bands were here not only present for the monocations of **4**, **5**, and **8**, but also for the higher oxidation states, and it is likely that some decompositions have occurred.

### UV–vis–NIR and EPR spectra of reduced species

**Figure 12:**
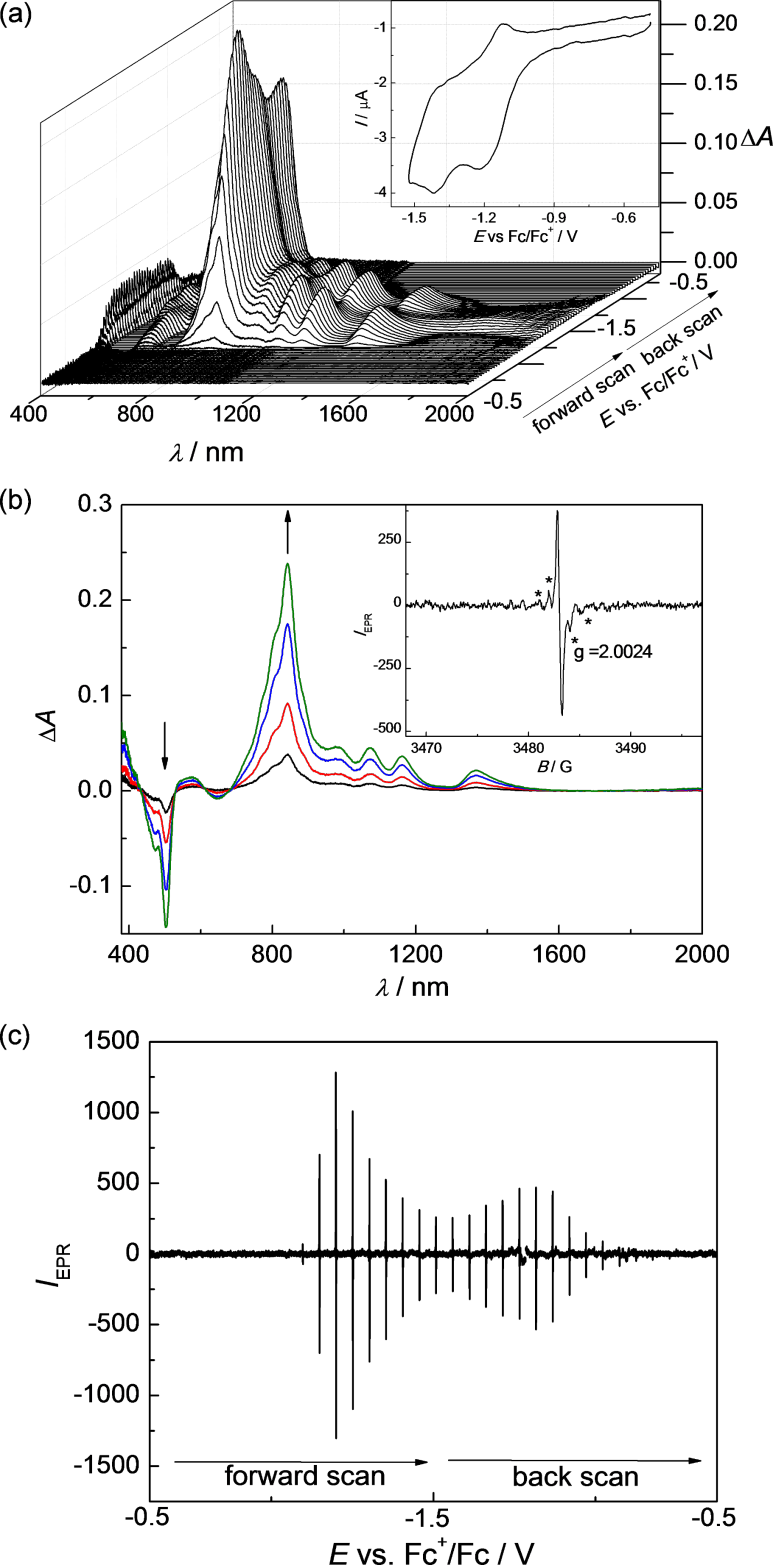
In situ EPR−UV–vis–NIR cyclic voltammetry of **2b** (1 mM) in the cathodic region: (a) potential dependence of vis–NIR spectra with corresponding cyclic voltammogram (in CH_2_Cl_2_ / 0.1 M Bu_4_PF_6_, scan rate *v* = 2 mV s^−1^); (b) representative difference vis–NIR spectra observed at the first reduction peak (inset: characteristic EPR spectrum of radical species monitored upon reduction); (c) potential dependence of EPR signal monitored during in situ cyclic voltammetric experiment.

In cyclic voltammetric studies discussed above, a nearly reversible first cathodic reduction was observed for radiaannulenes **2b** and **8**. At the low scan rates (2 mV s^−1^) used in spectroelectrochemistry, the cyclic voltammetric peaks become nearly irreversible indicating much lower stability of the formed radical anions in comparison to the corresponding radical cations. Nevertheless, a hint of counter peak in the back anodic scan at low scan rates for both compounds indicates that the primarily formed radical anions could be observed in the EPR spectroelectrochemical experiment. Figure 12 shows the vis–NIR/EPR spectrolectrochemical response found for sample **2b** in CH_2_Cl_2_ (Bu_4_NPF_6_) at a platinum mesh electrode. A new dominating absorption band with maximum at 842 nm having a rich vibronic pattern was observed during the in situ reduction in the region of the first cathodic voltammetric peak (a similar absorption at λ_max_ 845 nm was previously found for **2a**^•^**^–^** [10]) with the coincident monitoring of narrow single line EPR spectrum with *g*-value of 2.0024 (see Figure 12b). The intensity of the new optical band correlates well with the EPR intensity of the simultaneously-taken narrow EPR spectra (compare Figure 12a and 12c). However, in addition to the narrow EPR singlet line, an additional EPR signal was found (see EPR spectrum marked with asterisks in inset of Figure 12b), the intensity of which increased after prolonged reduction, indicating the formation of a new paramagnetic product by follow-up reactions. Consequently the first EPR signal can be tentatively ascribed to the radical anion **2b**^•^**^–^** while the second EPR signal is an unidentified product formed by conversion of this radical anion. Very similar EPR spectra were observed upon cathodic reduction of radiaannulene **8** as shown in Supporting Information File 1, Figure S13. A new optical band at λ_max_ = 653 nm accompanied with a narrow EPR signal at *g* = 2.0024 were monitored in the region of the first reduction peak. The observed *g*-values close to the free electron value for both anions **2b**^•^**^–^** and **8**^•^**^–^** are characteristic of delocalized spin and correspond to spin delocalization within the π_z_ aromatic core.

## Conclusion

In conclusion, the interactions between TTF units in dimeric structures depend strongly on the spacer unit in a rather complex manner. The interactions do not only rely on the length of the separation between the units, but also on the rigidity/planarity of the dimer, which can be enforced by cyclic cores. First of all, cyclic radiaannulene cores as spacers promote stepwise oxidation of each TTF unit to its radical cation, via an intermediate cationic species that exhibits an IVCT band. The generated dication is paramagnetic, signalling two unpaired TTF cation radicals. In contrast, TTF units separated by TEE-bridges in acyclic arrangements, either linearly conjugated or cross-conjugated, behaved as independent redox centres and the intermediate singly-charged *gem*-TEE species did not exhibit IVCT bands. With simple ethynediyl or butadiynediyl spacers in acyclic structures, each TTF unit was also oxidized almost at the same potential, but with some broadening of the first wave for the former compound and its monocation experienced a clear IVCT band, while this band was somewhat weaker for the monocation of the second one. An EPR silent species was generated for the dication of both of these compounds, which signals intramolecular interaction of the two polaronic TTF units as we exclude π-dimerization since the EPR inactivity did not depend on the concentration of the species. A non-negligible interaction between the polaronic states was observed for the dication of the radiaannulene with butadiynediyl and TEE-diyl-bridging units, but in this case, it did not lead to a spinless species. The CT absorptions observed for the radical cations with a short bridge between the two TTF units or a cyclic bridging unit are in general very weak, and it is difficult to make unambiguous assignments of these to intra- and/or intermolecular mixed valence transitions. It is noteworthy, however, that the dications (TTF^•+^–TTF^•+^) experienced Davydov redshifts rather than blueshifts, which signal intramolecular head-to-tail interactions rather than face-to-face π-dimer interactions when both TTF units exist as radical cations (in agreement with the paramagnetic properties). In addition, the triisopropylsilyl groups prevent formation of face-to-face dimers in which all TTFs interact pairwise for those radiaannulenes containing two such bulky groups, pointing to an intramolecular origin of the NIR absorptions.

Finally, the different radiaannulene cores also present redox-active units, undergoing reversible and ready reductions, which we ascribe to some gain in aromaticity upon reduction. On account of these electron-accepting properties, the neutral TTF-radiaannulenes exhibit low-energy CT absorptions.

## Experimental

**General Methods – Synthesis.** Chemicals were purchased from Aldrich, Fluka, and GSF Chemicals and used as received. THF was distilled over the Na/benzophenone couple. Solvents were of HPLC grade and used as received. Thin layer chromatography (TLC) was carried out using aluminum sheets precoated with silica gel 60 F_254_ (Merck 5554). Flash column chromatography was carried out using ROCC or Merck silica gel (40–63 μm). ^1^H NMR and ^13^C NMR spectra were recorded on a Bruker instrument (500/126 MHz) with non–inverse cryoprobe using the residual solvent as the internal standard (chloroform-*d* δ_H_ = 7.26 ppm, δ_C_ = 77.16 ppm; dichloromethane-*d*_2_ δ_H_ = 5.32 ppm, δ_C_ = 54.00 ppm). All coupling constants are expressed in Hertz (Hz). Melting points were measured on a Reichert melting point apparatus equipped with a microscope and are uncorrected. Mass spectrometry (MS) was performed using Matrix Assisted Laser Desorption Ionization (MALDI); TOF = time-of-flight; FT-ICR = Fourier Transform Ion Cyclotron Resonance. IR spectra were measured using the attenuated total reflectance (ATR) method on diamond. The relative peak intensities in IR spectra are designated as vw = very weak, w = weak, m = medium, s = strong.

**General methods – electrochemistry:** Cyclic voltammetry and differential pulse voltammetry were carried out in CH_2_Cl_2_ containing 0.1 M Bu_4_NPF_6_ as supporting electrolyte using Autolab PGSTAT12 or CH Instruments 400A or 630B potentiostats. The working electrode was a circular glassy carbon disk (*d* = 3 mm), the counter electrode was a platinum wire and the reference electrode was a silver wire immersed in the solvent-supporting electrolyte mixture and separated from the solution containing the substrate by a ceramic frit. The potential of the reference electrode was determined vs the ferrocene/ferrocenium (Fc/Fc^+^) redox system in a separate experiment. Solutions were purged with nitrogen saturated with CH_2_Cl_2_ for ten minutes before the measurements were made. Substrate concentrations were in all cases close to 1 mM. All measurements were carried out at room temperature (≈23 °C). **EPR/UV–vis–NIR spectroelectrochemistry:** Commercially available dichloromethane (CH_2_Cl_2_) and ferrocene (Fc) purchased from Sigma-Aldrich were used without further purification. Tetrabutylammonium hexafluorophosphate (Bu_4_NPF_6_) of puriss. quality (Fluka) was dried under reduced pressure at 70 °C for 24 h and stored in a desiccator. Cyclic voltammograms (CV) were recorded using a one-compartment electrochemical cell with platinum wires as working and counter electrodes and a Ag wire as a pseudo-reference electrode. Electrochemical measurements were performed under inert argon atmosphere on a PAR 273 potentiostat (EG&G, US) at room temperature. In situ ESR/UV–vis–NIR spectroelectrochemical experiments were performed in an optical ESR cavity (ER 4104OR, Bruker Germany) [34]. EPR spectra were recorded by an EMX X-band CW spectrometer (Bruker, Germany). UV–vis–NIR spectra were measured using the Avantes spectrometer AvaSpec-2048x14-USB2 with an CCD detector and AvaSpec-NIR256-2.2 with an InGaAs detector applying the AvaSoft 7.5 software. Both, the ESR spectrometer and the UV–vis–NIR spectrometer are linked to a HEKA potentiostat PG 390 which triggers both spectrometers. Triggering is performed by the software package PotMaster v2x40 (HEKA Electronik, Germany). For standard in situ ESR/vis–NIR spectroelectrochemical experiments an ESR flat cell was used. A laminated platinum mesh as the working electrode, a silver wire as the pseudo-reference electrode, and a platinum wire as the counter electrode were used in spectroelectrochemical experiments. To reach the nearly thin layer conditions, the electrolyte volume was reduced by inert foil sheets inserted into the flat cell. Experiments were also performed at room temperature in CH_2_Cl_2_ (0.1 M Bu_4_NPF_6_) using an optically transparent thin-layer electrochemical (OTTLE) cell equipped with a Pt mini grid working electrode (32 wires cm^–1^) and CaF_2_ windows [35]. The cell was positioned in the sample compartment with the photon source passing through the working electrode mini grid. A narrow slit width setting was chosen in the instrument settings to get good resolution. The UV–vis–NIR spectra were obtained using a Varian Cary 5E spectrophotometer in double beam mode. The controlled-potential electrolysis was carried out using a CH Instruments Model CHI630B potentiostat to manually adjust the potential.

**4',5'-Bis(butylthio)-4-(trimethylsilyl)tetrathiafulvalene (10).** To a degassed solution of **9** (315 mg, 0.62 mmol) in Et_3_N (15 mL) were added PdCl_2_(PPh_3_)_2_ (87 mg, 0.12 mmol), CuI (12 mg, 0.06 mmol) and trimethylsilylacetylene (0.70 mL, 5.0 mmol). The mixture was stirred at rt for 3 h after which it became orange. Petroleum spirit (10 mL) was added, the mixture was filtered through a short plug of silica (SiO_2_, CH_2_Cl_2_) and the solvent evaporated in vacuo. Column chromatography (SiO_2_, EtOAc/petroleum spirit 1:25) followed by size exclusion column chromatography (Biobeads, S-X3, CH_2_Cl_2_) afforded compound **10** (230 mg, 78%) as an orange oil. ^1^H NMR (500 MHz, CDCl_3_) δ 6.51 (s, 1H), 3.24–2.27 (m, 4H), 1.41 (p, *J* = 7.3 Hz, 4H), 1.32–1.15 (m, 4H), 0.72–0.52 (m, 6H), 0.00 (s, 9H) ppm; ^13^C NMR (126 MHz, CDCl_3_) δ 128.21, 127.66, 125.42, 116.12, 112.62, 109.26, 100.27, 94.97, 36.15, 36.14, 31.91, 31.89, 21.78, 13.74, −0.22 ppm (2 signals missing); MS (MALDI–TOF): *m*/*z* = 476 [M^•+^]; HRMS (MALDI+, FT-ICR, dithranol): *m/z* = 476.02777 [M^•+^] (calcd for C_19_H_28_S_6_Si^+^: 476.02790).

**1,2-Bis(4,5-bis(butylthio)tetrathiafulvalene)ethyne (4).** To a solution of K_2_CO_3_ (121 mg, 0.88 mmol) in MeOH (30 mL) was added a solution of **10** (56 mg, 0.12 mmol) in THF (5 mL). The mixture was stirred at rt for 20 min, until quantitative conversion was detected by TLC (SiO_2_, CH_2_Cl_2_/heptane 1:4). The mixture was diluted with Et_2_O (100 mL), washed with water (3 × 100 mL) and brine (3 × 100 mL). The organic phase was dried over MgSO_4_ and filtered. Et_3_N (15 mL) was added and the solution was concentrated in vacuo until only Et_3_N was left. The solution of the desilylated compound in Et_3_N was degassed and Pd(PPh_3_)_4_ (21 mg, 0.02 mmol) and CuI (7 mg, 0.04 mmol) were added followed by a degassed solution of **9** in Et_3_N (10 mL). After stirring at rt for 3 h, the color changed from orange to red; petroleum spirit was added and the mixture was filtered through a short plug of silica (SiO_2_ in CH_2_Cl_2_) and the solvent evaporated in vacuo. Size exclusion column chromatography (Biobeads, S-X3, CH_2_Cl_2_) gave compound **4** (73 mg, 78%) as a red amorphous solid. ^1^H NMR (500 MHz, CDCl_3_) δ 6.57 (s, 2H), 2.83–2.80 (m, 8H), 1.63–1.59 (m, 8H), 1.46–1.41 (m, 8H), 0.93 (t, *J* = 7.4 Hz, 6H), 0.92 (t, *J* = 7.4 Hz, 6H) ppm; ^13^C NMR (126 MHz, CDCl_3_) δ 128.28, 127.77, 126.50, 114.90, 111.75, 110.43, 84.61, 36.20, 36.19, 31.98, 31.91, 21.95, 13.75 ppm (2 signals missing); MS (MALDI–TOF): *m*/*z* = 782 [M^•+^]; HRMS (MALDI+, FT-IRC, dithranol): *m*/*z* = 781.96161 [M^•+^] (calcd. for C_30_H_38_S_12_^+^: 781.96165).

**1,4-Bis(4,5-bis(butylthio)tetrathiafulvalene)-1,3-butadiyne (5).** To a solution of K_2_CO_3_ (190 mg, 1.37 mmol) in MeOH (50 mL) was added a solution of **10** (164 mg, 0.34 mmol) in THF (5 mL). The mixture was stirred at rt for 20 min, until quantitative conversion was detected by TLC (SiO_2_, CH_2_Cl_2_/heptane 1:4). The mixture was diluted with CH_2_Cl_2_ (150 mL) and washed with brine (3 × 100 mL). The organic phase was dried over MgSO_4_, filtered and concentrated in vacuo until the total volume was ca. 50 mL. To the obtained solution of the desilylated product in CH_2_Cl_2_ were added CuCl (3 mg, 0.03 mmol), TMEDA (0.1 mL, 0.67 mmol), and 4 Å molecular sieves (0.12 g), instantly turning the mixture to red. The mixture was stirred vigorously for 2 h, after which it was passed through a short plug of neutralized silica (SiO_2_, CH_2_Cl_2_) and concentrated in vacuo. Size exclusion column chromatography (Biobeads, S-X3, CH_2_Cl_2_) gave compound **5** (77 mg, 56%) as a red amorphous solid. ^1^H NMR (500 MHz, CDCl_3_) δ 6.70 (s, 2H), 2.83–2.79 (m, 8H), 1.63–1.57 (m, 8H), 1.47–1.40 (m, 8H), 0.92 (t, *J* = 7.4 Hz, 6H), 0.92 (t, *J* = 7.4 Hz, 6H) ppm; ^13^C NMR (126 MHz, CDCl_3_) δ 129.61, 128.34, 127.70, 114.89, 111.27, 111.20, 77.92, 75.31, 36.19, 31.92, 31.89, 21.79, 13.74 ppm (3 signals missing); MS (MALDI–TOF): *m*/*z* = 806 [M^•+^]; HRMS (MALDI+, FT-IRC, dithranol): *m*/*z* = 805.96170 [M^•+^] (calcd. for C_32_H_38_S_12_^+^: 805.96165).

***trans*-TEE-TTF (6).** A solution of **11** (37 mg, 0.09 mmol) in (iPr)_2_NH (8 mL) was degassed with argon on an ultrasound bath for 30 min. Then Pd(PPh_3_)_4_ (7 mg, 0.01 mmol), CuI (4 mg, 0.02 mmol), followed by **9** (106 mg, 0.21 mmol) in degassed (iPr)_2_NH (2 mL) were added, instantly turning the mixture to dark purple. The mixture was protected from light and stirred at rt for 4 h. Heptane (20 mL) was added and the mixture was filtered through a short plug of silica (SiO_2_, CH_2_Cl_2_) and concentrated in vacuo. Column chromatography (SiO_2_, heptane) afforded compound **6** (90 mg, 89%) as a dark purple oil. ^1^H NMR (500 MHz, CD_2_Cl_2_) δ 6.63 (s, 2H), 2.86–2.79 (m, 8H), 1.75–1.54 (m, 8H), 1.51–1.39 (m, 8H), 1.23–1.12 (m, 42H), 1.03–0.82 (m, 12H); ^13^C NMR (126 MHz, CD_2_Cl_2_) δ 128.76, 128.19, 127.75, 117.30, 115.75, 112.42, 110.47, 105.11, 102.61, 92.01, 90.10, 36.59, 32.42, 32.37, 22.20, 22.19, 19.08, 13.92, 11.87 ppm (2 signals missing); HRMS (MALDI+, FT-ICR, dithranol): *m/z* = 1192.24515 [M^•+^] (calcd for C_56_H_80_S_12_Si_2_^+^: 1192.24415).

**Compound 7.** A flask containing Pd(PPh_3_)_4_ (11 mg, 0.01 mmol), 2,6-bis(ethynyl)pyridine (6.3 mg, 0.05 mmol), and CuI (1 mg, 0.01 mmol) was charged with an argon balloon. A solution of **9** (50 mg, 0.01 mmol) in argon-flushed Et_3_N (10 mL) was added and the resulting orange reaction mixture was stirred for 6 h. The reaction was quenched by addition of saturated aqueous NH_4_Cl (10 mL) followed by water (100 mL), and the mixture was extracted with CH_2_Cl_2_ (100 mL). The organic extract was washed with saturated aqueous NH_4_Cl (3 × 50 mL), dried with Na_2_SO_4_, filtered and concentrated in vacuo. Purification by flash column chromatography (SiO_2_ pre-treated with a 2% solution of Et_3_N, CH_2_Cl_2_/petroleum spirit 1:9 to 1:4, loaded using CCl_4_), followed by size-exclusion chromatography (Biobeads S-X3, CH_2_Cl_2_) gave compound **7** (35 mg, 80%) as a red oil which slowly solidified. M.p. 91–93 °C (CH_2_Cl_2_). ^1^H NMR (400 MHz, CDCl_3_) δ 7.65 (t, *J* = 7.8 Hz, 1H), 7.38 (d, *J* = 7.8 Hz, 2H), 6.71 (s, 2H), 2.82 (t, *J* = 7.3 Hz, 4H), 2.81 (t, *J* = 7.3 Hz, 4H), 1.64–1.60 (m, 8H), 1.47–1.41 (m, 8H), 0.93 (t, *J* = 7.3 Hz, 6H), 0.93 (t, *J* = 7.3 Hz, 6H) ppm; ^13^C NMR (126 MHz, CDCl_3_) δ 143.01, 136.78, 128.29, 127.80, 127.65, 126.78, 115.05, 111.99, 110.21, 91.89, 80.97, 36.20, 31.94, 31.92, 21.81, 21.80, 13.76, 13.75 ppm (1 signal missing); HRMS (MALDI+, FT-ICR, dithranol) *m*/*z* = 882.99032 [M^•+^] (calcd. for C_37_H_41_N^+^: 882.98820).

**Compound 14.** A solution of **13** (99 mg, 0.07 mmol) in (iPr)_2_NH (10 mL) was degassed on an ultrasound bath for 30 min. Thereafter, PdCl_2_(PPh_3_)_2_ (5 mg, 0.01 mmol), CuI (1 mg, 0.01 mmol), and trimethylsilylacetylene (0.15 mL, 1.03 mmol) were subsequently added. After stirring at rt for 1.5 h, the mixture was filtered through a plug of silica (SiO_2_, CH_2_Cl_2_/petroleum spirit 1:1) and concentrated in vacuo to give compound **14** (49 mg, 52%) as a green oil. ^1^H NMR (500 MHz, CDCl_3_) δ 2.81 (t, *J* = 7.3 Hz, 4H), 2.81 (t, *J* = 7.3 Hz, 4H), 1.64–1.58 (m, 8H), 1.46–1.41 (m, 8H), 1.12–1.11 (m, 42H), 0.92 (t, *J* = 7.3 Hz, 6H), 0.92 (t, *J* = 7.3 Hz, 6H), 0.22 (s, 18H) ppm; ^13^C NMR (126 MHz, CDCl_3_) δ 127.92, 127.87, 122.84, 120.88, 119.92, 115.19, 112.03, 109.43, 107.77, 105.30, 103.51, 95.07, 94.51, 89.23, 36.22, 31.94, 31.92, 21.80, 18.88, 13.75, 11.41, −0.24 ppm (3 signals missing); HRMS (MALDI+, FT-ICR, dithranol): *m*/*z* = 1384.32641 [M^•+^] (calcd. for C_66_H_96_S_12_Si_4_^+^: *m*/*z* = 1384.32321).

**Radiaannulene 8.** To a solution of K_2_CO_3_ (30 mg, 0.22 mmol) in MeOH (20 mL) was added a solution of **14** (74 mg, 0.05 mmol) in THF (5 mL). The mixture was stirred at rt for 20 min, until quantitative conversion was detected by TLC (SiO_2_, CH_2_Cl_2_/heptane 1:4). The mixture was diluted with CH_2_Cl_2_ (150 mL) and washed with brine (3 × 100 mL). The organic phase was dried over MgSO_4_, filtered and concentrated in vacuo until the total volume was ca. 50 mL. To the obtained solution of the desilylated product in CH_2_Cl_2_ were added CuCl (2 mg, 0.02 mmol), TMEDA (0.1 mL, 0.67 mmol), and 4 Å molecular sieves (0.06 g). The mixture was stirred vigorously for 2 h, after which it was passed through a short plug of silica (SiO_2_, CH_2_Cl_2_) and concentrated in vacuo. Size exclusion column chromatography (Biobeads, S-X3, CH_2_Cl_2_) gave compound **8** (24 mg, 37%) as a green oil. Crystals suitable for X-ray crystallography were grown from CH_2_Cl_2_/MeOH. ^1^H NMR (500 MHz, CDCl_3_) δ 2.83–2.80 (m, 8H), 1.64–1.60 (m, 8H), 1.46–1.41 (m, 8H), 1.15–1.14 (m, 42H), 0.93 (t, *J* = 7.4 Hz, 6H), 0.93 (t, *J* = 7.4 Hz, 6H) ppm; ^13^C NMR (126 MHz, CDCl_3_) δ 128.34, 127.75, 127.59, 121.79, 120.68, 114.29, 108.45, 108.28, 103.90, 98.13, 91.56, 87.85, 86.92, 36.32, 36.28, 31.99, 31.91, 21.82, 21.79, 18.93, 13.74, 13.73, 11.48 ppm (1 signal missing); IR (ATR): 2955s, 2927s, 2863s, 2725vw, 2154w (C≡C), 1716vw, 1676vw, 1461m, 1417w, 1380w, 1346w, 1254m, 1224w, 1201w cm^−1^; HRMS (MALDI+, FT-ICR, dithranol): *m*/*z* = 1238.22912 [M^•+^] (calcd. for C_60_H_78_S_12_Si_2_^+^: *m*/*z* = 1238.22851).

## Supporting Information

File 1 UV–vis absorption spectra, electrochemical data, UV–vis–NIR absorption spectra of oxidized species, EPR spectra, NMR spectra, and computational data.

File 2 CIF-file of X-ray crystal structure **8**.

## References

[1] Adam M, Müllen K (1994). Adv Mater.

[2] Iyoda M, Hasegawa M, Miyake Y (2004). Chem Rev.

[3] Lorcy D, Bellec N, Formigué M, Avarvari N (2009). Coord Chem Rev.

[4] Lahlil K, Moradpour A, Bowlas C, Menou F, Cassoux P, Bonvoisin J, Launay J-P, Dive G, Dehareng D (1995). J Am Chem Soc.

[5] Otsubo T, Kochi Y, Bitoh A, Ogura F (1994). Chem Lett.

[6] Iyoda M, Hasegawa M, Takano J-i, Hara K, Kuwatani Y (2002). Chem Lett.

[7] Hara K, Hasegawa M, Kuwatani Y, Enozawa H, Iyoda M (2004). Chem Commun.

[8] Andersson A S, Kerndrup L, Madsen A Ø, Kilså K, Nielsen M B, La Porta P R, Biaggio I (2009). J Org Chem.

[9] Lincke K, Christensen M A, Diederich F, Nielsen M B (2011). Helv Chim Acta.

[10] Lincke K, Frellsen A F, Parker C R, Bond A D, Hammerich O, Nielsen M B (2012). Angew Chem, Int Ed.

[11] Parthey M, Gluyas J B, Schauer P A, Yufit D S, Howard J A K, Kaupp M, Low P J (2013). Chem – Eur J.

[12] Parthey M, Gluyas J B G, Fox M A, Low P J, Kaupp M (2014). Chem – Eur J.

[13] Low P J, Roberts R L, Cordiner R L, Hartl F (2005). J Solid State Electrochem.

[14] Hasegawa M, Kobayashi Y, Hara K, Enozawa H, Iyoda M (2009). Heterocycles.

[15] Mazzanti V, Jiang H, Gotfredsen H, Morsing T J, Parker C R, Nielsen M B (2014). Org Lett.

[16] Hasegawa M, Enozawa H, Kawabata Y, Iyoda M (2007). J Am Chem Soc.

[17] Vilhelmsen M H, Jensen J, Tortzen C G, Nielsen M B (2013). Eur J Org Chem.

[18] Anthony J, Boudon C, Diederich F, Gisselbrecht J-P, Gramlich V, Gross M, Hobi M, Seiler P (1994). Angew Chem, Int Ed.

[R19] 19Please notice that the current values given for **2a** in Figure 6 of [10] by a mistake are a factor of 1000 too small.

[20] Iyoda M, Kuwatani Y, Ueno N, Oda M (1992). J Chem Soc, Chem Commun.

[21] Iyoda M, Fukuda M, Yoshida M, Sasaki S (1994). Chem Lett.

[22] Vacher A, Barrière F, Roisnel T, Piekara-Sady L, Lorcy D (2011). Organometallics.

[23] Misaki Y, Matsui T, Kawakami K, Nishikawa H, Yamabe T, Shiro M (1993). Chem Lett.

[24] Dembinski R, Bartik T, Bartik B, Jaeger M, Gladysz J A (2000). J Am Chem Soc.

[25] Bruce M I, Cole M L, Ellis B G, Gaudio M, Nicholson B K, Parker C R, Skelton B W, White A H (2015). Polyhedron.

[26] Lapinte C (2008). J Organomet Chem.

[27] Parker V D, Tilset M, Hammerich O (1987). J Am Chem Soc.

[28] Khodorkovsky V, Shapiro L, Krief P, Shames A, Mabon G, Gorgues A, Giffard M (2001). Chem Commun.

[29] Kirketerp M-B S, Leal L A E, Varsano D, Rubio A, Jørgensen T J D, Kilså K, Nielsen M B, Nielsen S B (2011). Chem Commun.

[30] Hasegawa M, Daigoku K, Hashimoto K, Nishikawa H, Iyoda M (2012). Bull Chem Soc Jpn.

[31] Rosokha S V, Kochi J K (2007). J Am Chem Soc.

[32] Rapta P, Lukkari J, Tarábek J, Salomäki M, Jussila M, Yohannes G, Riekkola M-L, Kankare J, Dunsch L (2004). Phys Chem Chem Phys.

[33] van Haare J A E H, Havinga E E, van Dongen J L J, Janssen R A J, Cornil J, Brédas J-L (1998). Chem – Eur J.

[34] Matis M, Rapta P, Lukeš V, Hartmann H, Dunsch L (2010). J Phys Chem B.

[35] Krejčík M, Daněk M, Hartl F (1991). J Electroanal Chem.

