# Supplementary material for: Interactions between tetrathiafulvalene units in dimeric structures – the influence of cyclic cores
Source: Beilstein J Org Chem. 2015 Jun 2;11:930–48. doi: 10.3762/bjoc.11.104 (PMC4464444; doi:10.3762/bjoc.11.104)
Supplement: File 1 — UV–vis absorption spectra, electrochemical data, UV–vis–NIR absorption spectra of oxidized species, EPR spectra, NMR spectra, and computational data. [file Beilstein_J_Org_Chem-11-930-s001.pdf]

Supporting Information

for

Interactions between tetrathiafulvalene units in dimeric structures – the influence of cyclic cores

Huixin Jiang[1], Virginia Mazzanti[1,2], Christian R. Parker[1], Søren Lindbæk Broman[1], Jens Heide Wallberg[1], Karol Lušpai[3], Adam Brincko[3], Henrik G. Kjaergaard[1], Anders Kadziola[1], Peter Rapta[3]*, Ole Hammerich[1]* and Mogens Brøndsted Nielsen[1]*

Address: [1]Department of Chemistry, University of Copenhagen, Universitetsparken 5, DK-2100 Copenhagen Ø, Denmark, [2]Sino-Danish Centre for Education and Research (SDC), Niels Jensens Vej 2, DK-8000 Aarhus C, Denmark, and [3]Institute of Physical Chemistry and Chemical Physics, Faculty of Chemical and Food Technology, Slovak University of Technology, Radlinskeho 9, 81237 Bratislava, Slovak Republic

Email:Peter Rapta - peter.rapta@stuba.sk; Ole Hammerich - o.hammerich@chem.ku.dk; , Mogens Brøndsted Nielsen - mbn@kiku.dk

*Corresponding author

UV–vis absorption spectra, electrochemical data, UV–vis–NIR absorption spectra of oxidized species, EPR spectra, NMR spectra, and computational data.

**UV–vis absorption spectra ……………………………………………..**	S2
**Electrochemistry ……………………………………………………….**	S4
**EPR / UV–vis–NIR spectroelectrochemistry ………….…………….**	S9
**UV–vis–NIR spectroelectrochemistry ……..……………………….**	S16
**NMR spectra …………………………………………………………….**	S20
**IR spectrum of 8 ……………………………………………………….**	S27
**Calculations …………………………………………………………….**	S28

UV–vis absorption spectra

Experimental

Compounds **1b** and **4–8** were dissolved in commercially available dichloromethane (CH_2Cl_2) from Labscan and transferred to a quartz cuvette with a 1 cm path length. UV–vis spectra were recorded on a Cary 5000 spectrometer in a 200–3000 nm wavelength region with a step size of 1 nm and a 2 nm slit width. The detectors used were a PMT and a PbS in the 200–800 nm region and 800–3000 nm region, respectively. A deuterium light source was used in the 200-300 nm region and a tungsten halogen light source in the 300–3000 nm region. The spectra were recorded in double beam mode with a cuvette containing CH_2Cl_2 in the reference beam. λ_{max}-values and the corresponding ε_{max}-values were found using the Peak Analyzer tool from OriginPro 9.1 using the Local Maximum method. The peaks were filtered by number by increasing the number until all peaks had been identified.

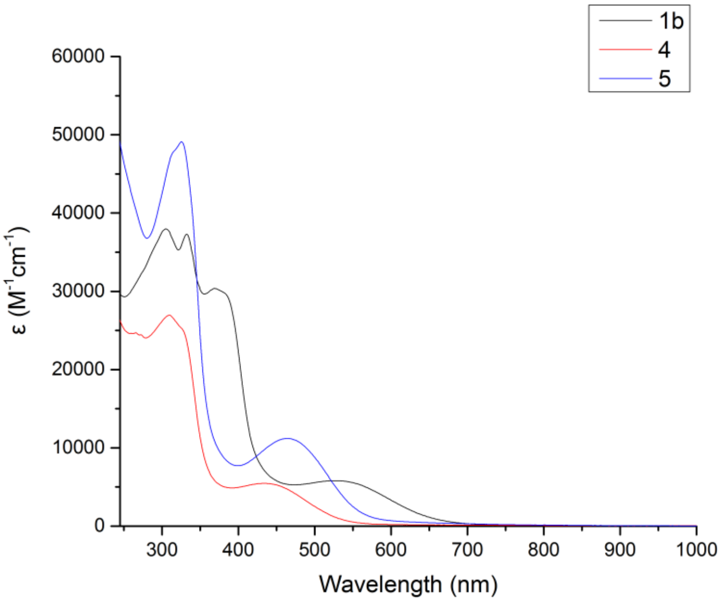

Figure S1: UV–vis absorption spectra of compounds **1b**, **4**, and **5** recorded in CH_2Cl_2 solution.

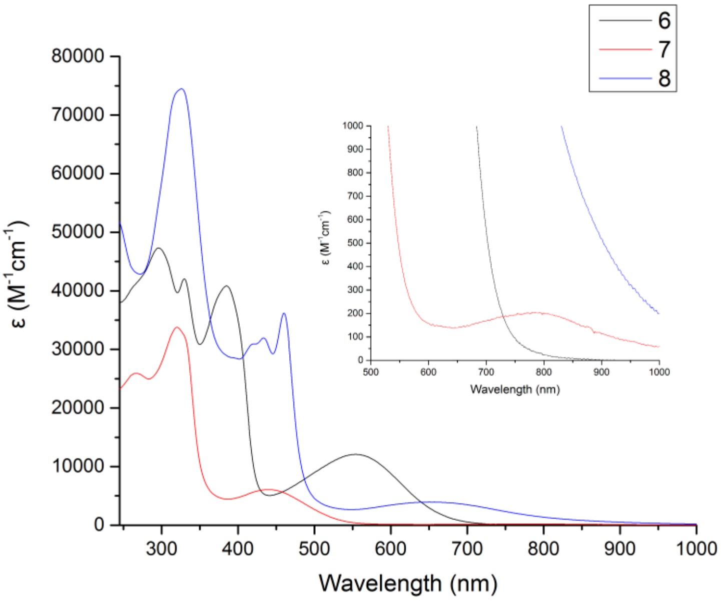

Figure S2: UV–vis absorption spectra of compounds **6**, **7**, and **8** recorded in CH_2Cl_2 solution, including a close-up of the 500–1000 nm.

Table S1. Absorption maxima (λ_{max} / nm) and the corresponding extinction coefficients (ε_{max} / $M^{-1}cm^{-1}$) of compounds in CH_2Cl_2.

	λ_{max}^{1}	ε_{max}^{1}	λ_{max}^{2}	ε_{max}^{2}	λ_{max}^{3}	ε_{max}^{3}	λ_{max}^{4}	ε_{max}^{4}
**1b**	305	3.8×10^{4}	333	3.7×10^{4}	369	3.0×10^{4}	529	5.8×10^{3}
**4**	266	2.5×10^{4}	310	2.7×10^{4}	435	5.5×10^{3}	-	-
**5**	326	4.9×10^{4}	465	1.1×10^{4}	-	-	-	-
**6**	296	4.7×10^{4}	330	4.2×10^{4}	385	4.1×10^{4}	554	1.2×10^{4}
**7**	267	2.6×10^{4}	320	3.4×10^{4}	439	6.1×10^{3}	779	2.1×10^{2}
**8**	326	7.4×10^{4}	434	3.2×10^{4}	460	3.6×10^{4}	651	3.9×10^{3}

Electrochemistry

Differential pulse voltammograms – oxidations

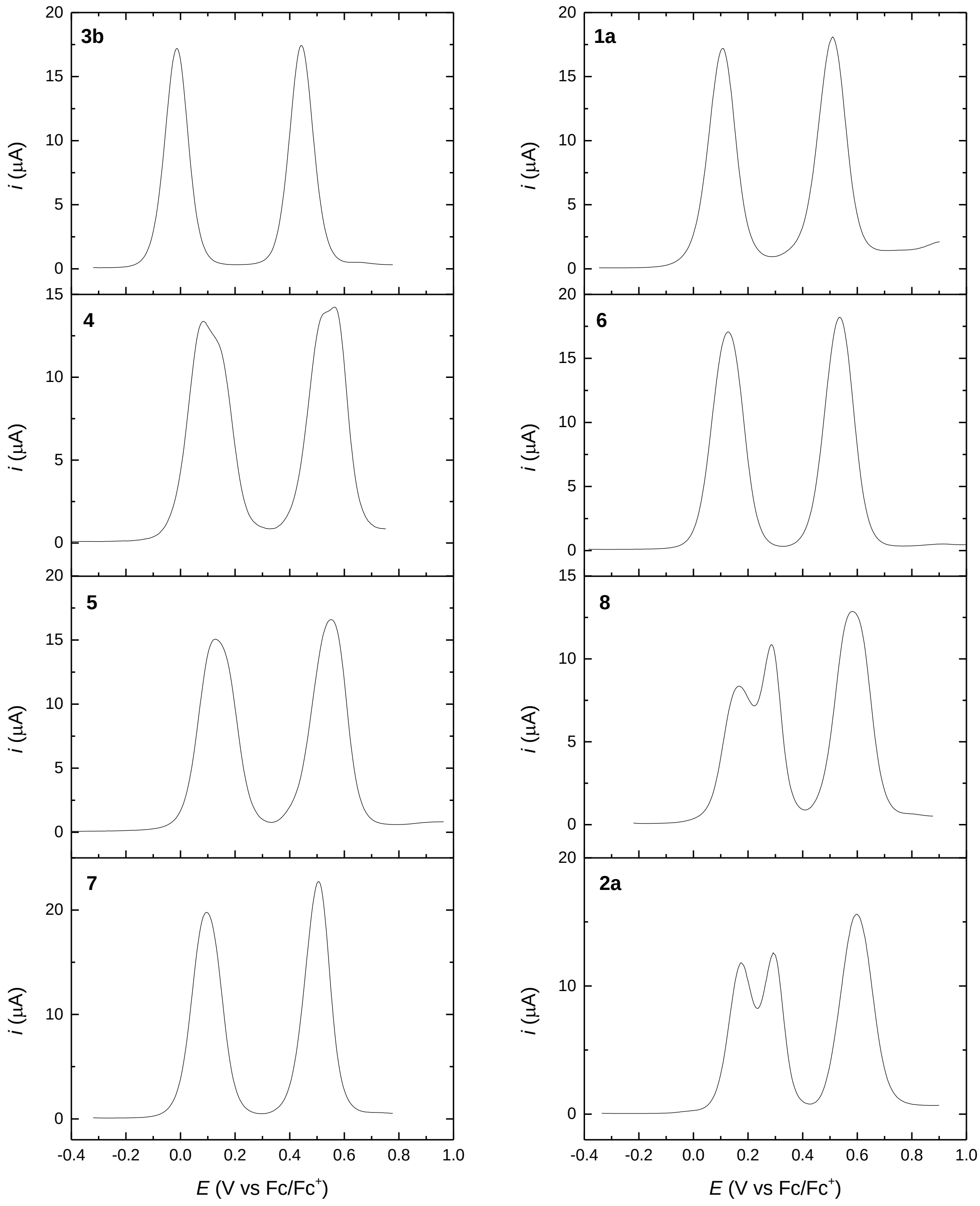

Figure S3: Results obtained by differential pulse voltammetry (DPV) for the oxidation of compounds **1a**, **2a**, **3b**, **4–8** in CH_2Cl_2 (0.1 M Bu_4NPF_6) at a glassy carbon electrode. Step potential: 0.0045 V. Modulation amplitude: 0.025 V.

Cyclic voltammograms – oxidations

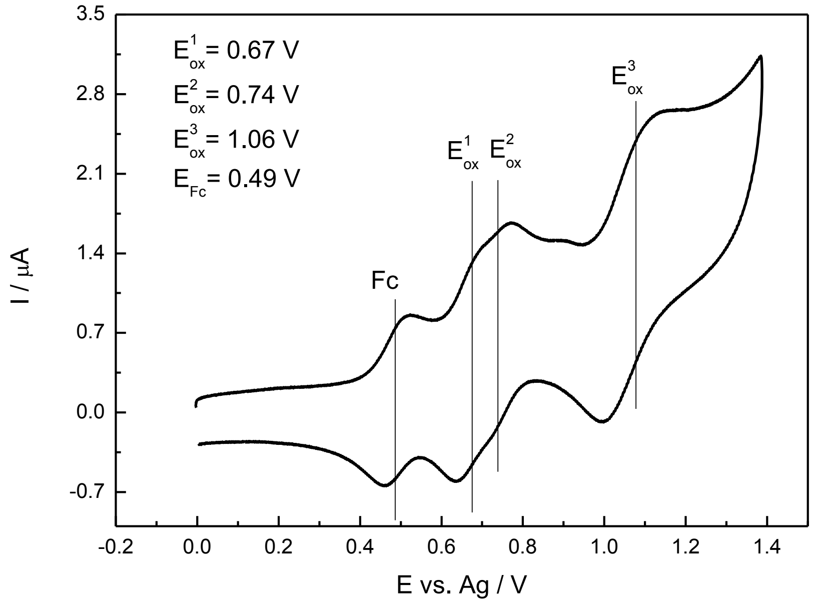

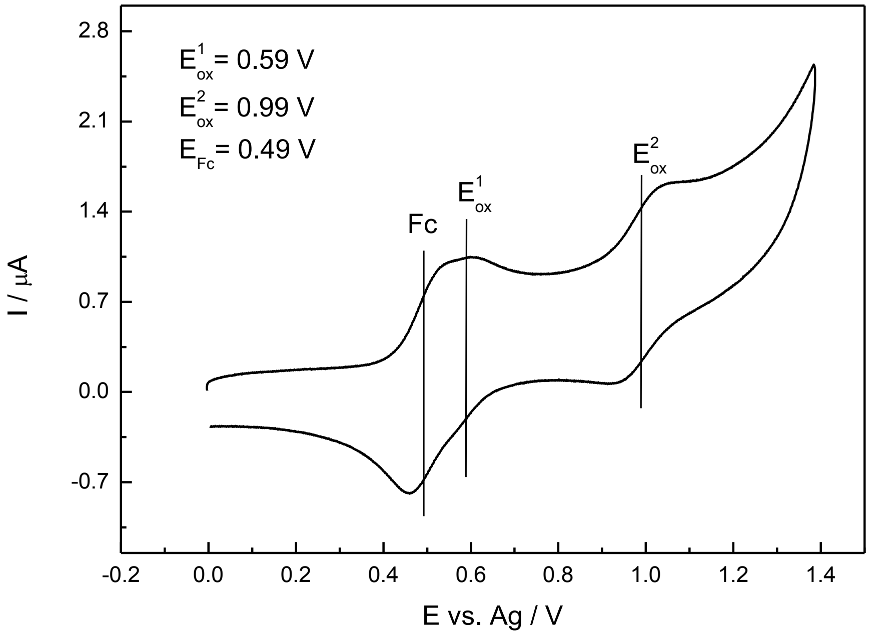

Figure S4: Cyclic voltammetry of **2b** and **1b** in CH_2Cl_2 (0.1 M Bu_4NPF_6) at a platinum-wire working electrode with a scan rate of 0.1 V s^{-1} in the presence of internal ferrocene potential marker.

Reductions

Table S2: Electrochemical data for the reduction of compounds **1–2, 4–8**.[a]

	Cyclic Voltammetry (CV)			Differential Pulse Voltammetry (DPV)		
	E_{red}^{1} / V	E_{red}^{2} / V	E_{red}^{3} / V	E_{p}^{1} / V	E_{p}^{2} / V	E_{p}^{3} / V
**1a**	-1.65	-2.15[b]		-1.61	-2.08	
**2a**	-1.16	-1.52		-1.12	-1.47	
**4**	-2.5[b,c]			-2.36		
**5**	-2.35[c,d]			-2.12		
**6**	-1.65	-2.18[c]	-2.37[c,f]	-1.60	-2.01	-2.25[f]
**7**	-2.30/-2.40[b,e]			-2.22		
**8**	-1.12	-1.51		-1.11	-1.55	

[a]Recorded at a glassy carbon working electrode in CH_2Cl_2 (0.1 M Bu_4NPF_6), potentials are given *vs* Fc/Fc^+. CV scan rate: 0.1 V s^{-1}. DPV step potential: 0.0045 V. DPV modulation amplitude: 0.025 V.[b]Shoulder on the backgound. [c]Irreversible peak. [d]Broad peak. [e]Double peak. [f]Product peak.

Differential pulse voltammograms – reductions

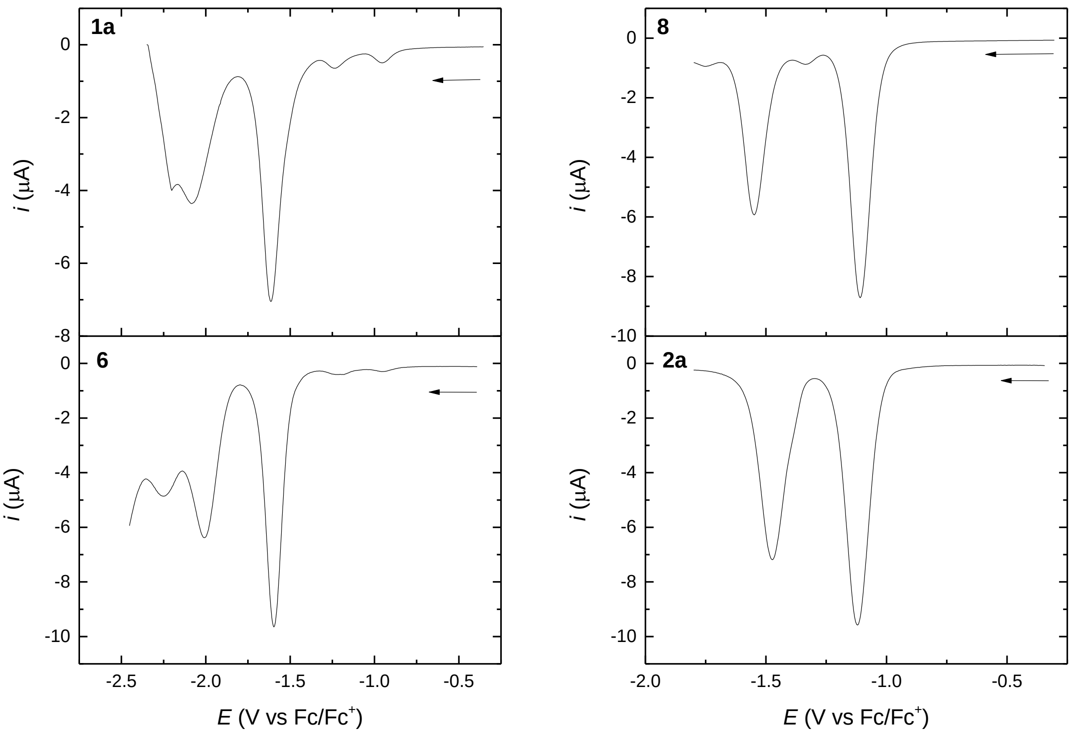

Figure S5: Results obtained by differential pulse voltammetry for the reduction of compounds **1a**, **2a**, **6** and **8** in CH_2Cl_2 (0.1 M Bu_4NPF_6) at a glassy carbon electrode. DPV step potential: 0.0045 V. DPV modulation amplitude: 0.025 V.

Cyclic and differential pulse voltammograms – reductions

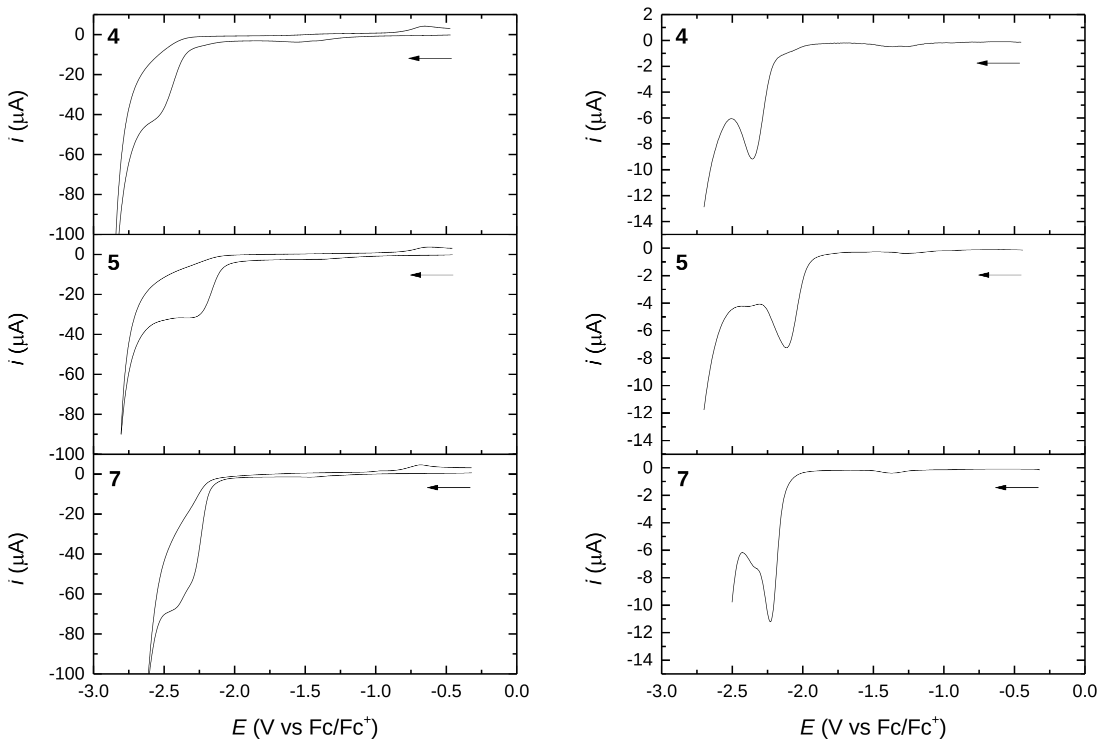

Figure S6: Results obtained by cyclic voltammetry (left) and differential pulse voltammetry (right) for the reduction of compounds **4**, **5**, and **7** in CH_2Cl_2 (0.1 M Bu_4NPF_6) at a glassy carbon electrode. CV scan rate: 0.1 V s^{-1}. DPV step potential: 0.0045 V. DPV modulation amplitude: 0.025 V.

EPR / UV–vis–NIR Spectroelectrochemistry

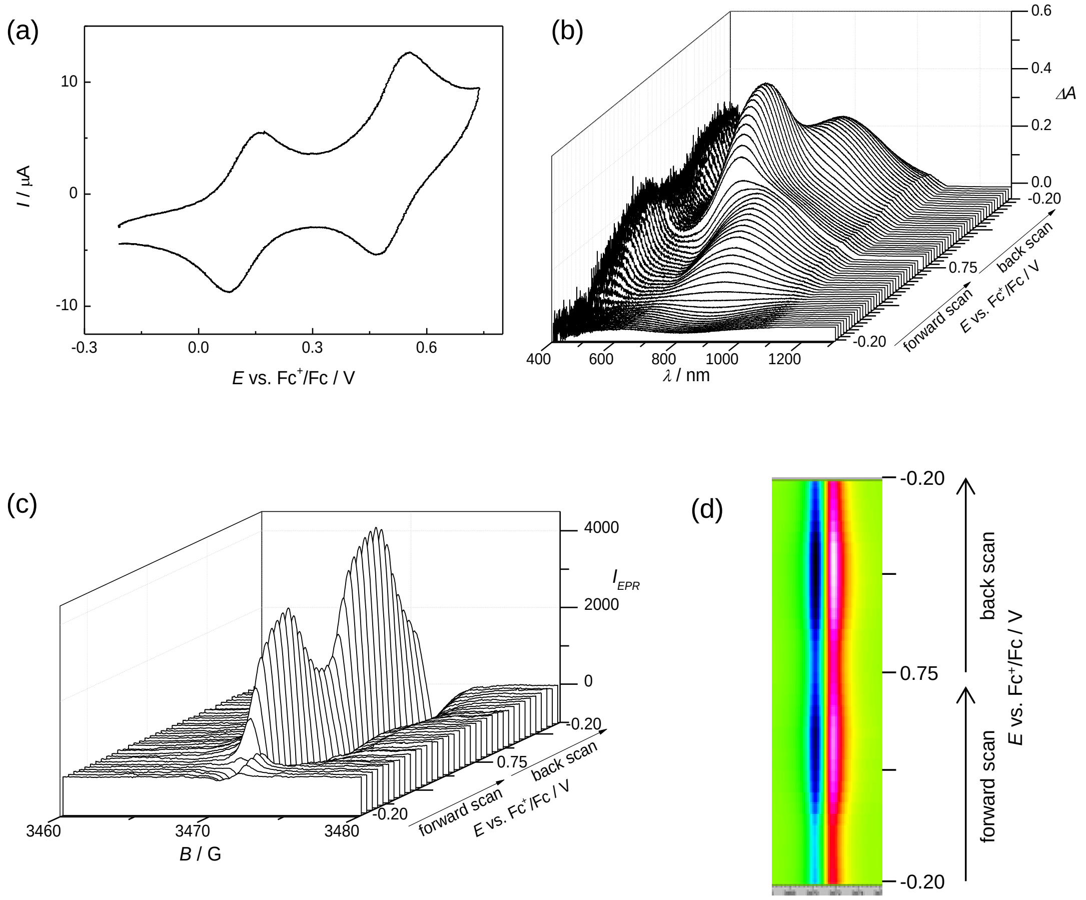

Figure S7: In situ EPR−UV/vis/NIR cyclic voltammetry of **1b**: (a) corresponding cyclic voltammogram in CH_2Cl_2 / 0.1 M Bu_4PF_6 (scan rate v = 2 mV s^{-1}); (b) potential dependence of vis/NIR spectra; (c) potential dependence of EPR spectra and (d) 2D EPR density plot of EPR signal observed during the in situ EPR/UV–vis–NIR cyclic voltammetry.

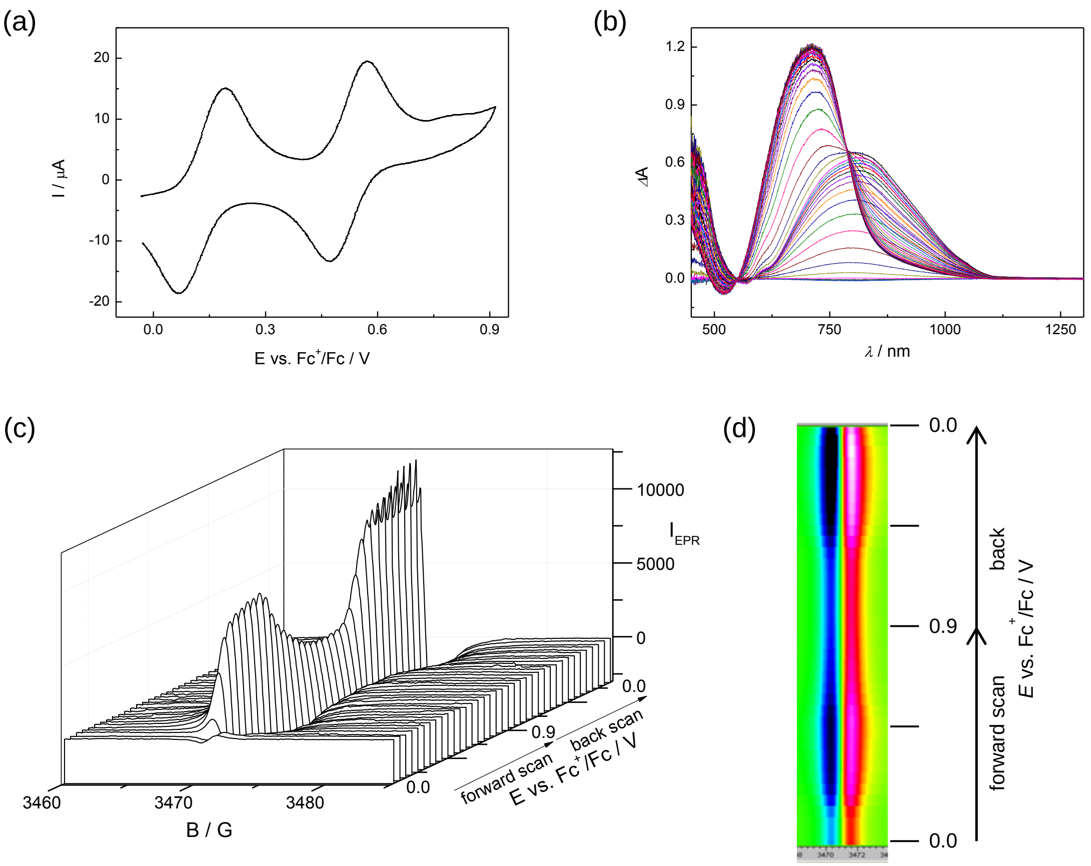

Figure S8: In situ EPR−UV/vis/NIR cyclic voltammetry of **6**: (a) corresponding cyclic voltammogram in CH_2Cl_2 / 0.1 M Bu_4PF_6 (scan rate v = 2 mV s^{-1}); (b) evolution of vis/NIR spectra in 2D projection; (c) potential dependence of EPR spectra and (d) 2D EPR density plot of EPR signal observed during the in situ EPR/UV–vis–NIR cyclic voltammetry.

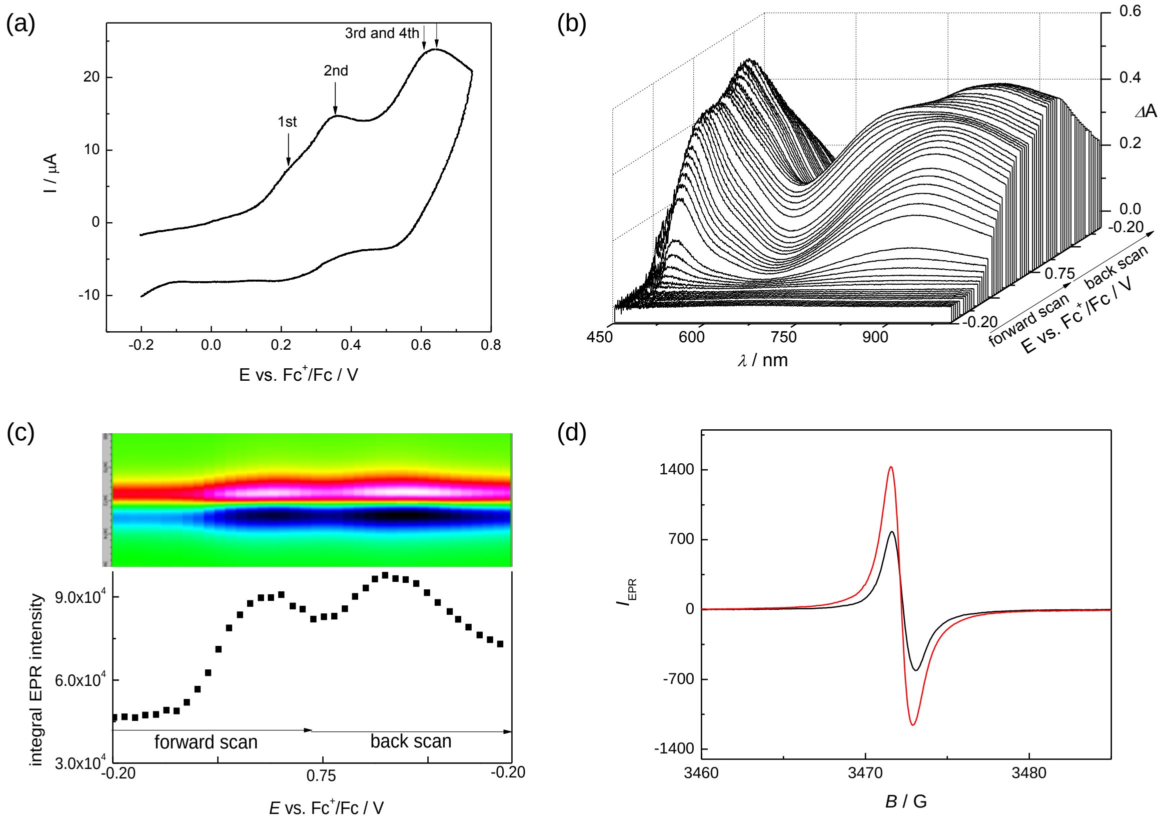

Figure S9: In situ EPR−UV/vis/NIR cyclic voltammetry of **8**: (a) corresponding cyclic voltammogram in CH_2Cl_2 / 0.1 M Bu_4PF_6 (scan rate v = 2 mV s^{-1}); (b) evolution of vis/NIR spectra in 3D projection; (c) potential dependence of EPR integral intensity (inset above: 2D EPR density plot of EPR signal observed during the in situ cyclic voltammetry); (d) representative EPR spectra observed at the first (black line) and at the second (red line) oxidation step.

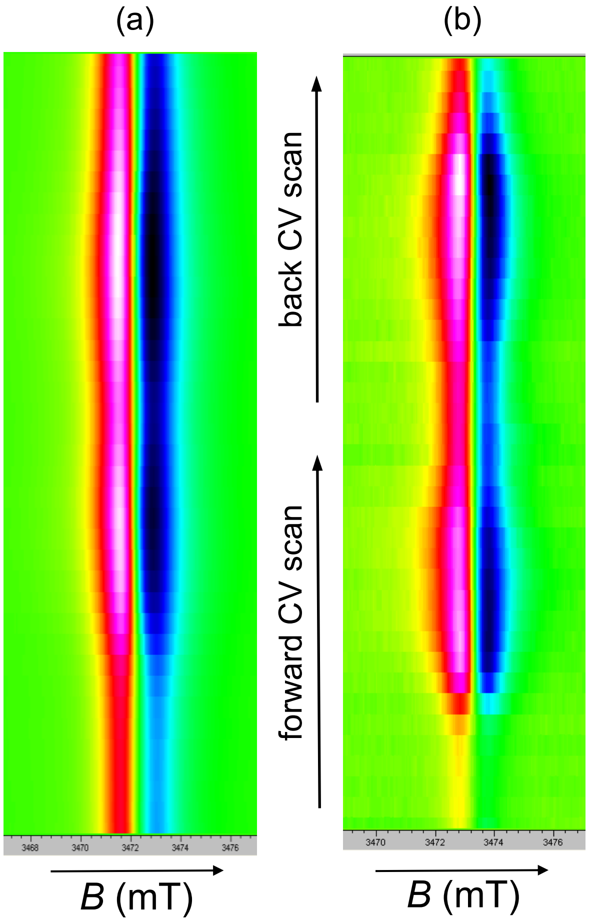

Figure S10: 2D ESR density plot of EPR signal observed during the in situ EPR/cyclic voltammetry for (a) 1 mM sample **8** and (b) 0.05 mM sample **8** in 0.1 M Bu_4NPF_6 in CH_2Cl_2 (scan rate 2 mV s^{-1}) in the region of both anodic voltammetric double peaks.

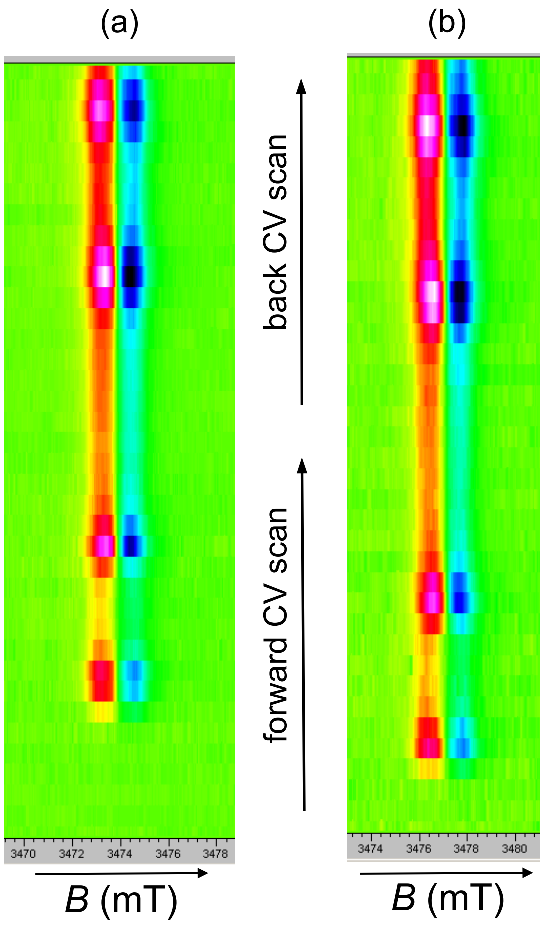

Figure S11: 2D ESR density plot of EPR signal observed during the *in situ* EPR/cyclic voltammetry for (a) 0.4 mM sample **4** and (b) 0.05 mM sample **4** in 0.1 M Bu_4NPF_6 in CH_2Cl_2 (scan rate 2 mV s^{-1}) in the region of both anodic voltammetric double peaks.

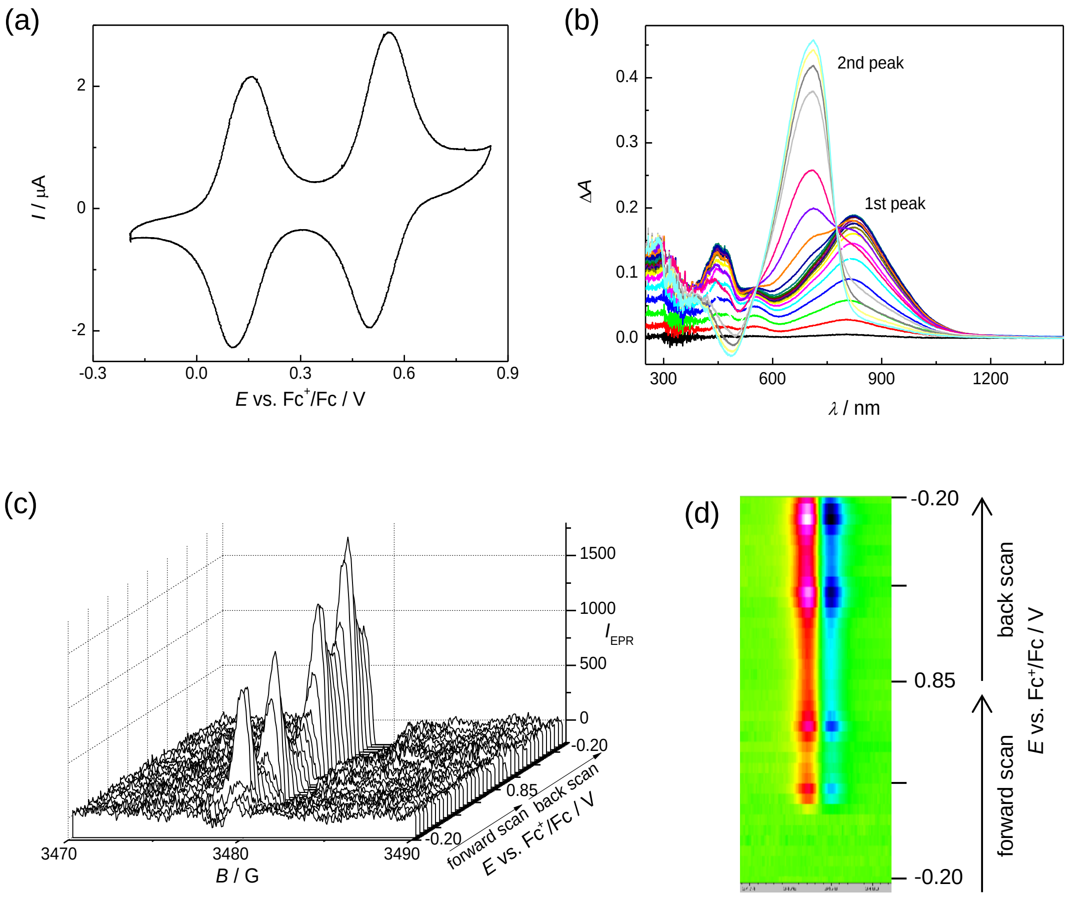

Figure S12: In situ EPR−UV/vis/NIR cyclic voltammetry of sample **5**: (a) corresponding cyclic voltammogram in CH_2Cl_2 / 0.1 M Bu_4PF_6 (scan rate v = 2 mV s^{-1}); (b) evolution of vis/NIR spectra in forward scan in 2D projection; (c) potential dependence of EPR spectra and (d) 2D EPR density plot of EPR signal observed during the *in situ* EPR/UV–vis–NIR cyclic voltammetry.

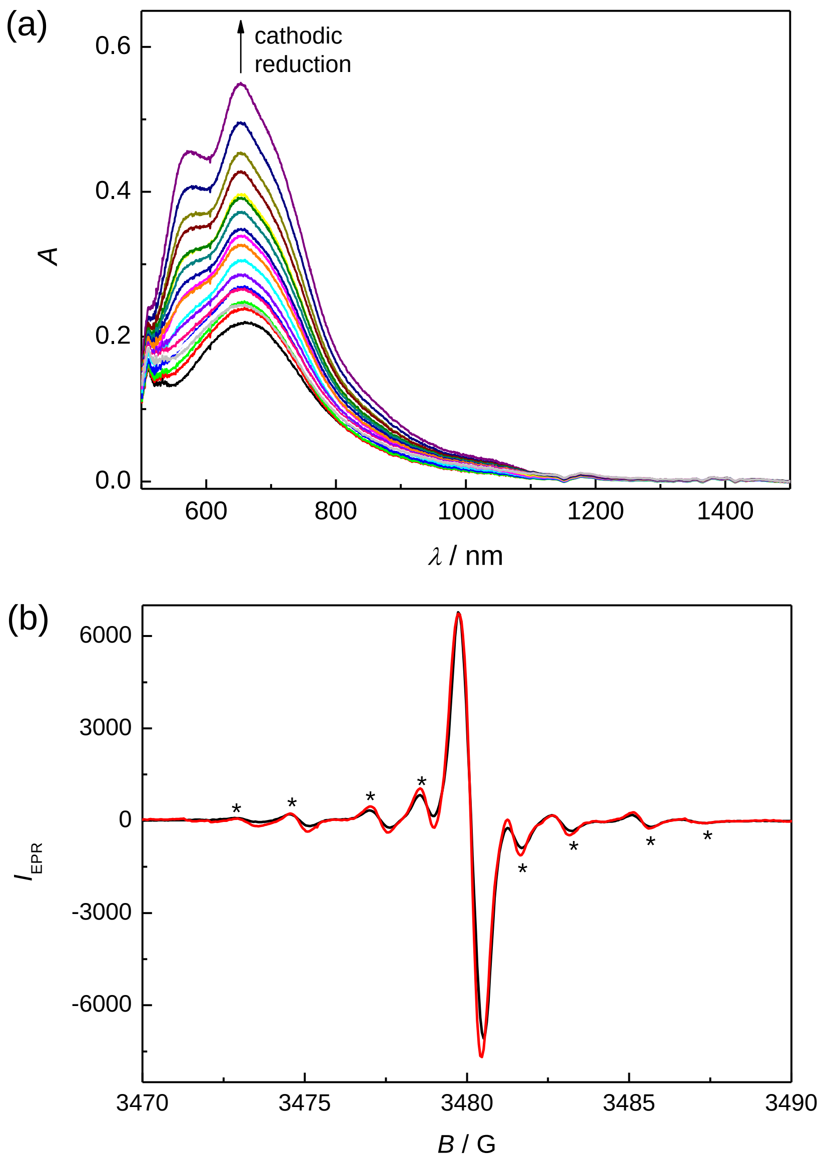

Figure S13: (a) Vis–NIR spectra monitored upon cathodic reduction at the maximum of the first cathodic peak of sample **8** in CH_2Cl_2 / 0.1 M Bu_4PF_6. (b) Representative EPR spectra observed in the initial stages of reduction (black line) and after prolonged reduction (red line) normalised to the maximal intensity of the central narrow singlet EPR line (EPR line of the additional radical is marked with asterisks).

UV–vis–NIR spectroelectrochemistry

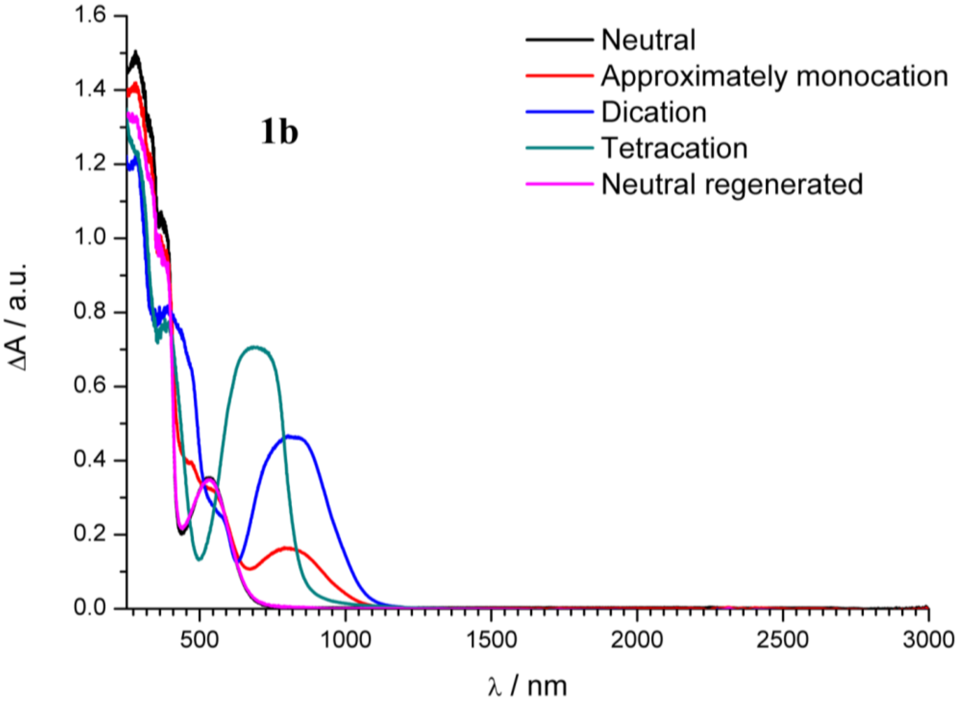

Figure S14: UV–vis–NIR absorption spectra of **1b** in CH_2Cl_2 + 0.1 M Bu_4NPF_6 at different oxidation states (obtained by electrolysis in an Ottle cell).

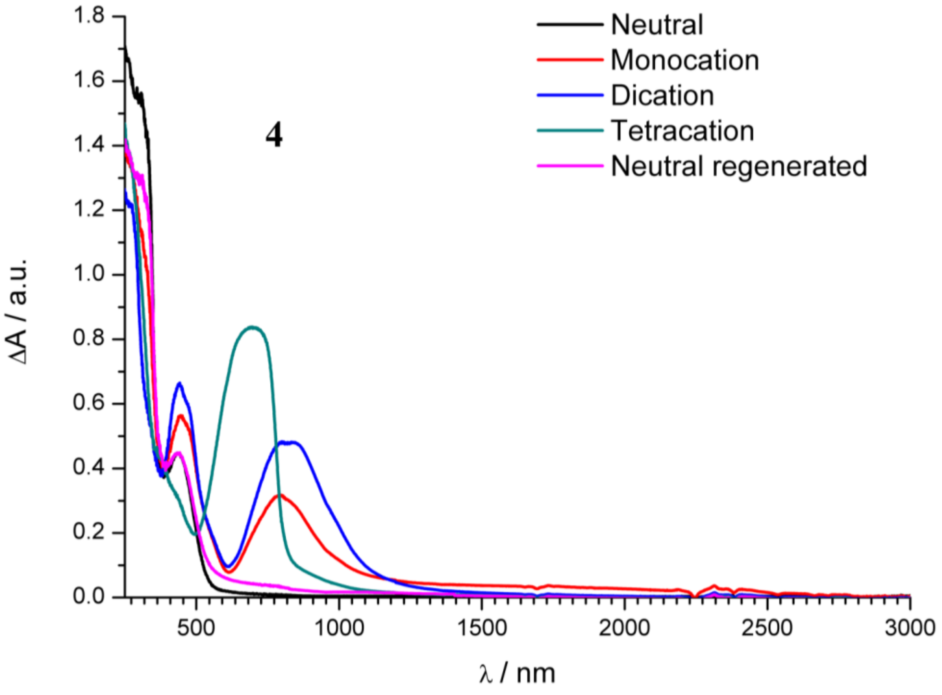

Figure S15: UV–vis–NIR absorption spectra of **4** in CH_2Cl_2 + 0.1 M Bu_4NPF_6 at different oxidation states (obtained by electrolysis in an Ottle cell). The spectrum assigned “Neutral regenerated” corresponds almost to the spectrum of the neutral compound, but it has not been fully reached yet at this stage of the electrolysis.

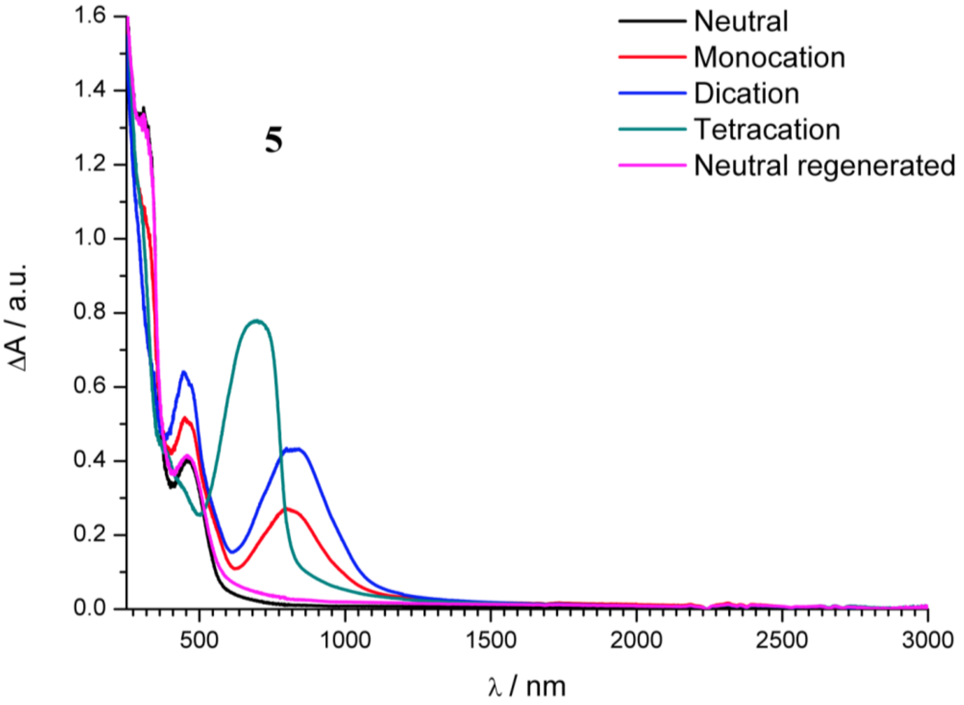

Figure S16: UV–vis–NIR absorption spectra of **5** in CH_2Cl_2 + 0.1 M Bu_4NPF_6 at different oxidation states (obtained by electrolysis in an Ottle cell).

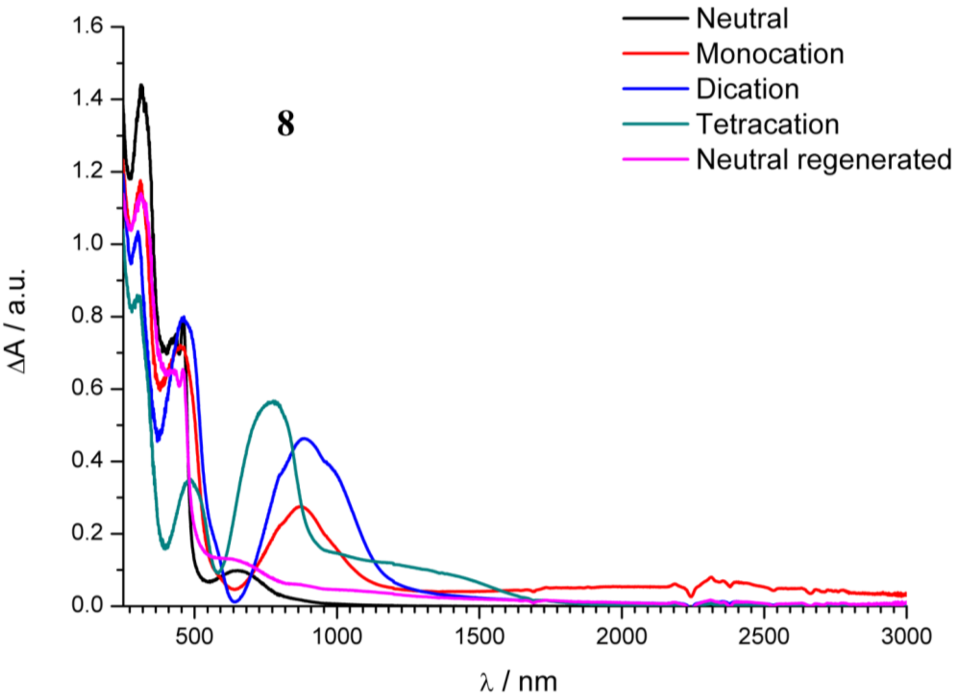

Figure S17: UV–vis–NIR absorption spectra of **8** in CH_2Cl_2 + 0.1 M Bu_4NPF_6 at different oxidation states (obtained by electrolysis in an Ottle cell). The spectrum of the regenerated neutral compound was not fully identical to the original one; thus, some degradation seems to have occurred.

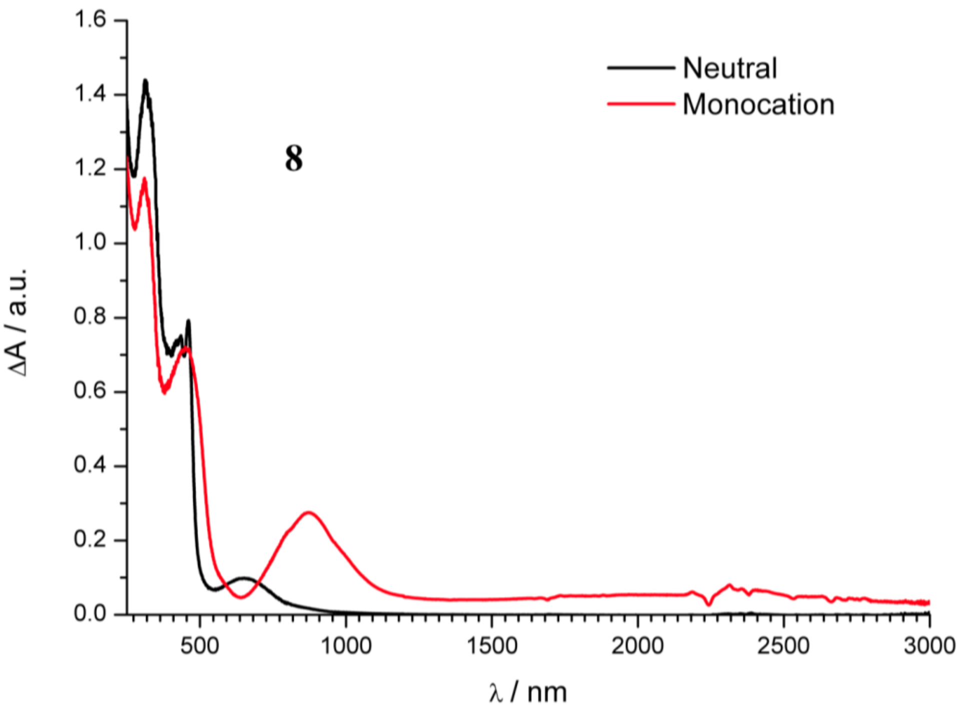

Figure S18: UV–vis–NIR absorption spectra of **8** and $\mathbf{8}^{\bullet+}$ in CH_2Cl_2 + 0.1 M Bu_4NPF_6 at different oxidation states (obtained by electrolysis in an Ottle cell).

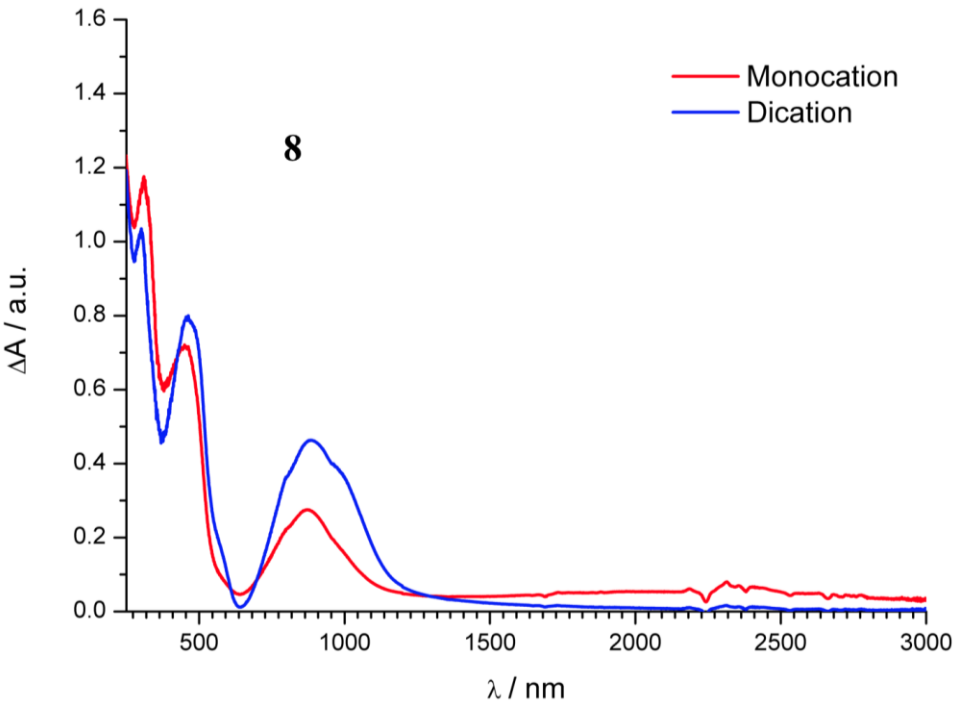

Figure S19: UV–vis–NIR absorption spectra of $\mathbf{8}^{\bullet+}$ and $\mathbf{8}^{2+}$ in CH_2Cl_2 + 0.1 M Bu_4NPF_6 at different oxidation states (obtained by electrolysis in an Ottle cell).

Table S3. Absorption maxima (λ_{max} / nm) of neutral and oxidized species in CH_2Cl_2 + 0.1 M Bu_4NPF_6.

Compound	λ_{max} / nm
**1b**	282, 321 (sh) , 365 (sh), 534
"**1b**$^{\bullet+}$"	278, 331 (sh), 359 (sh), 471(sh), 536 (sh), 811
**1b**$^{2+}$	281, 359 (sh), 579(sh), 821
**1b**$^{4+}$	266 (sh), 353, 687
**4**	307 (sh), 442
**4**$^{\bullet+}$	271 (sh), 446, 797, 1000-2500 (br)
**4**$^{2+}$	273 (sh), 440, 821
**4**$^{4+}$	269 (sh), 693
**5**	308 (sh), 464
**5**$^{\bullet+}$	316 (sh), 454, 803
**5**$^{2+}$	446, 825
**5**$^{4+}$	303 (sh), 375 (sh), 694
**8**	311, 434 (sh), 461, 648
**8**$^{\bullet+}$	309, 453, 870, 1500-3000 (br)
**8**$^{2+}$	302, 464, 886
**8**$^{4+}$	307, 486, 775

NMR spectra

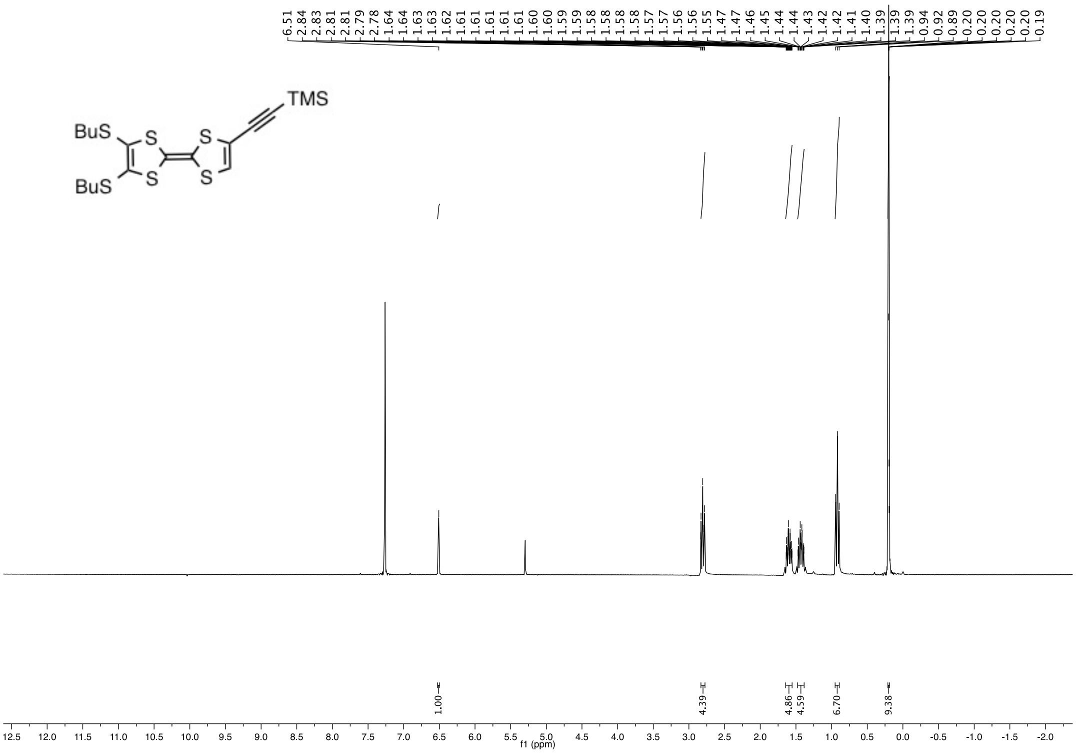

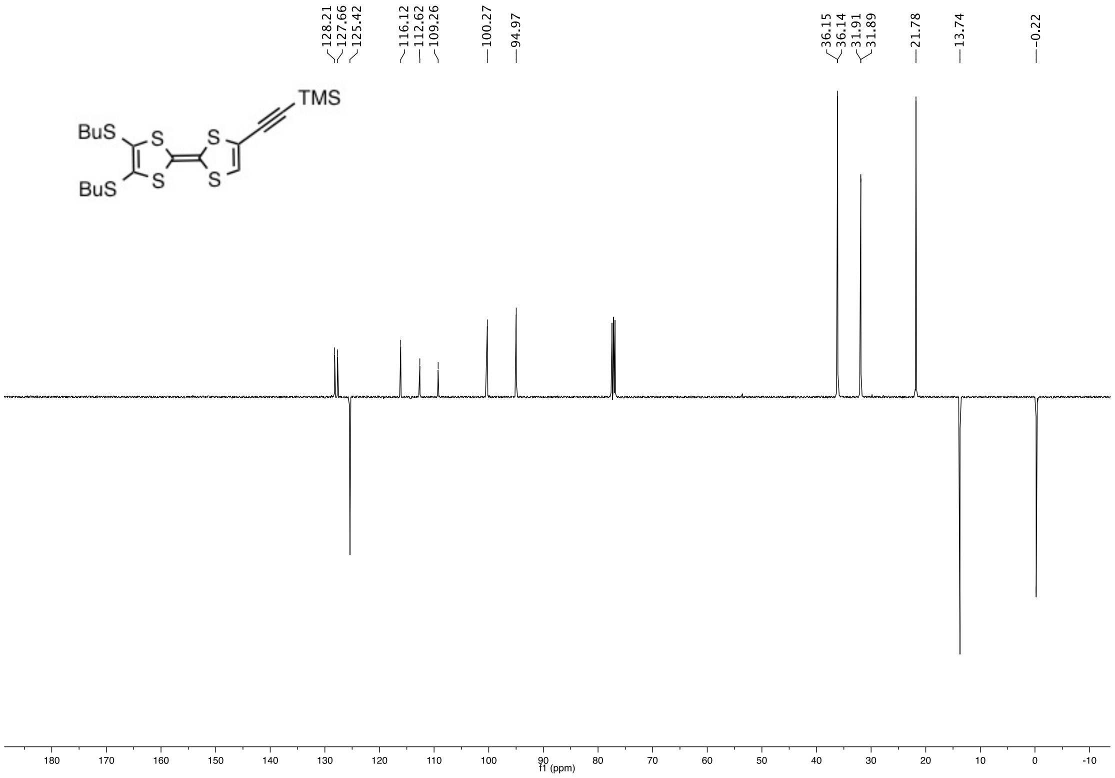

Figure S20: ^{1}H- and APT ^{13}C-NMR spectra of compound **10**.

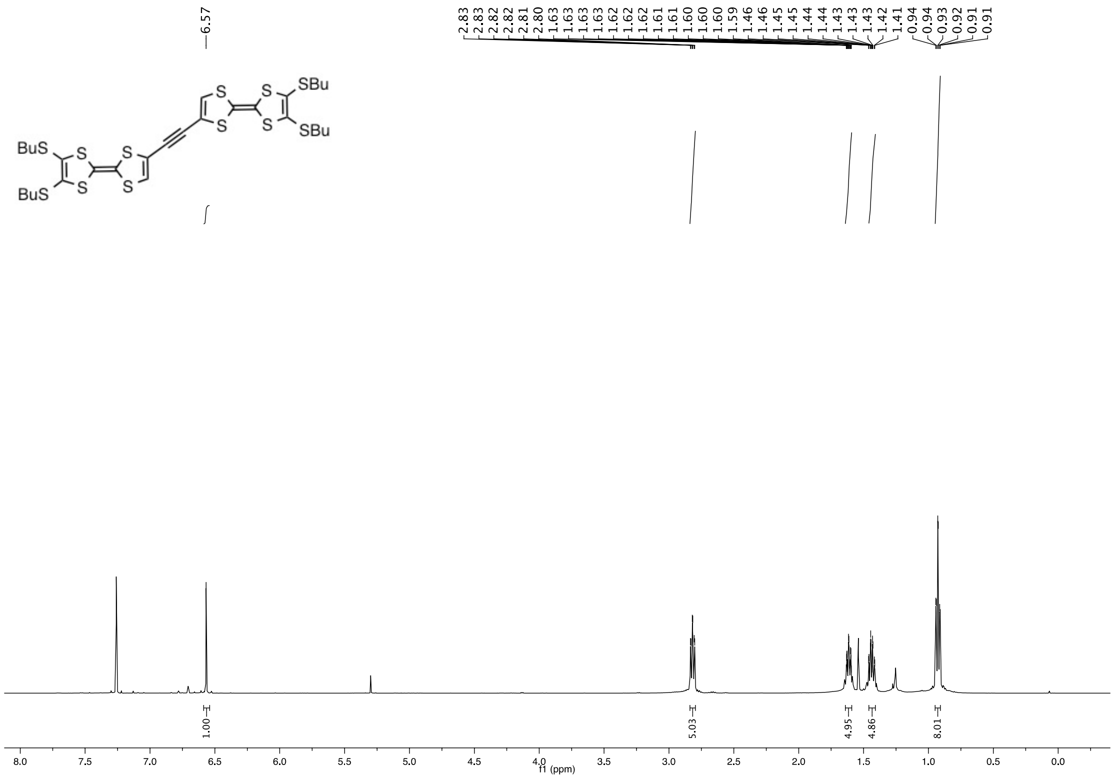

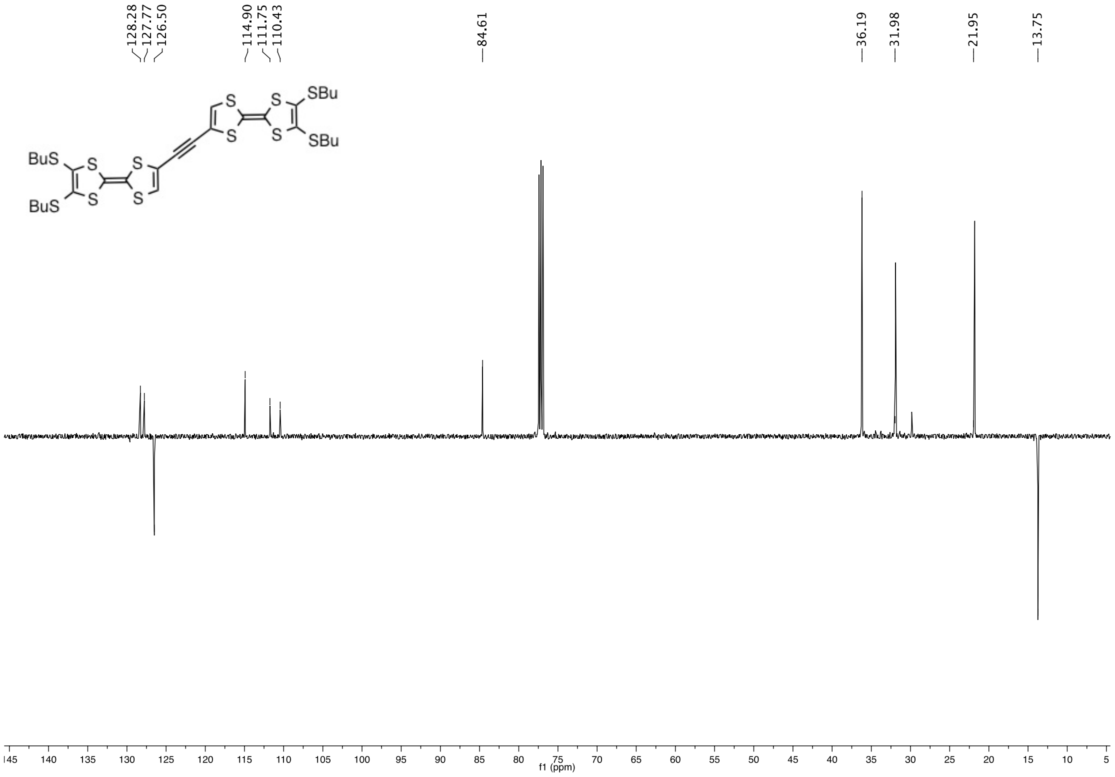

Figure S21: ^{1}H- and APT ^{13}C-NMR spectra of compound **4**.

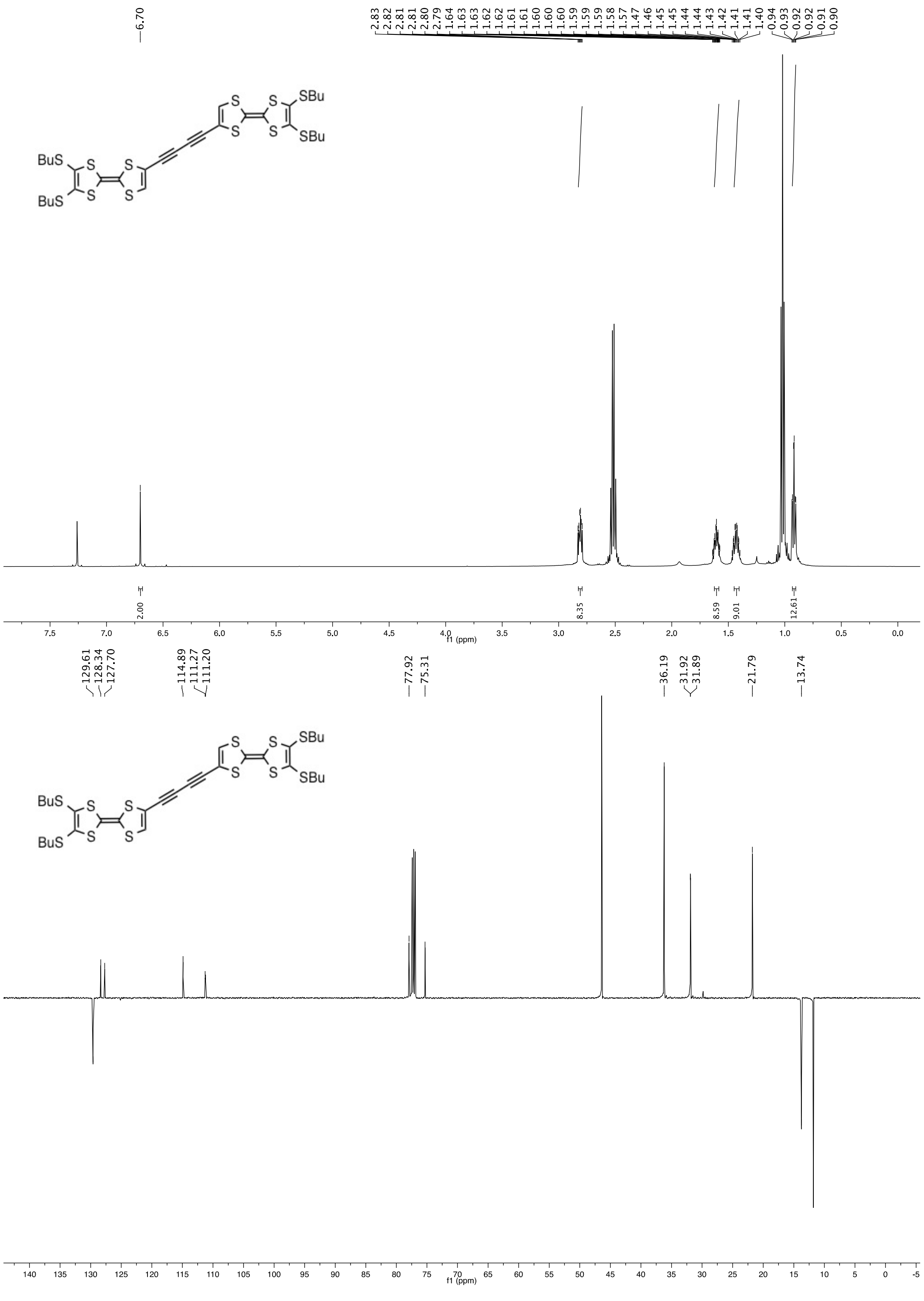

Figure S22: ^{1}H- and APT ^{13}C-NMR spectra of compound **5**.

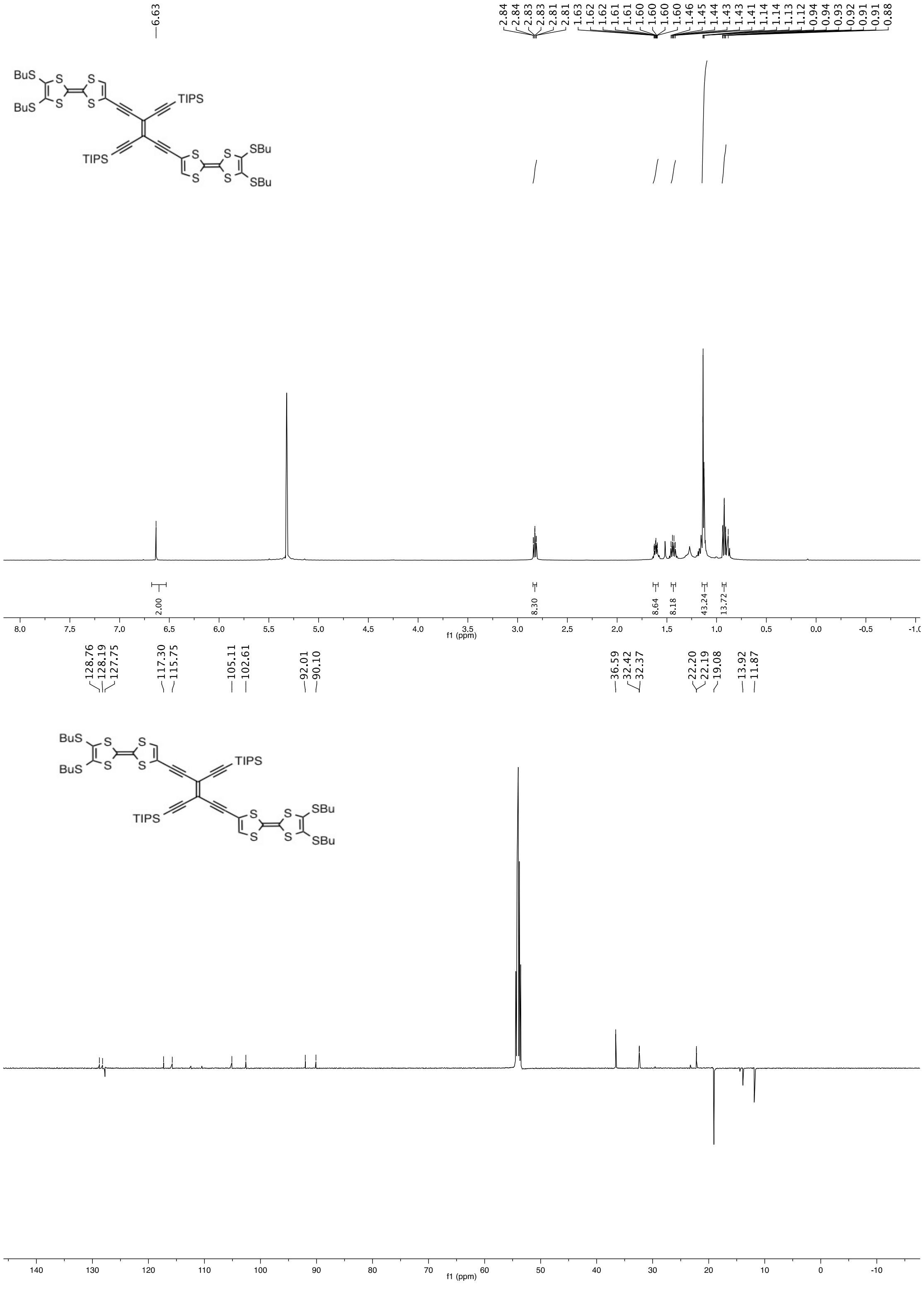

Figure S23: ^{1}H- and APT ^{13}C-NMR spectra of compound **6**.

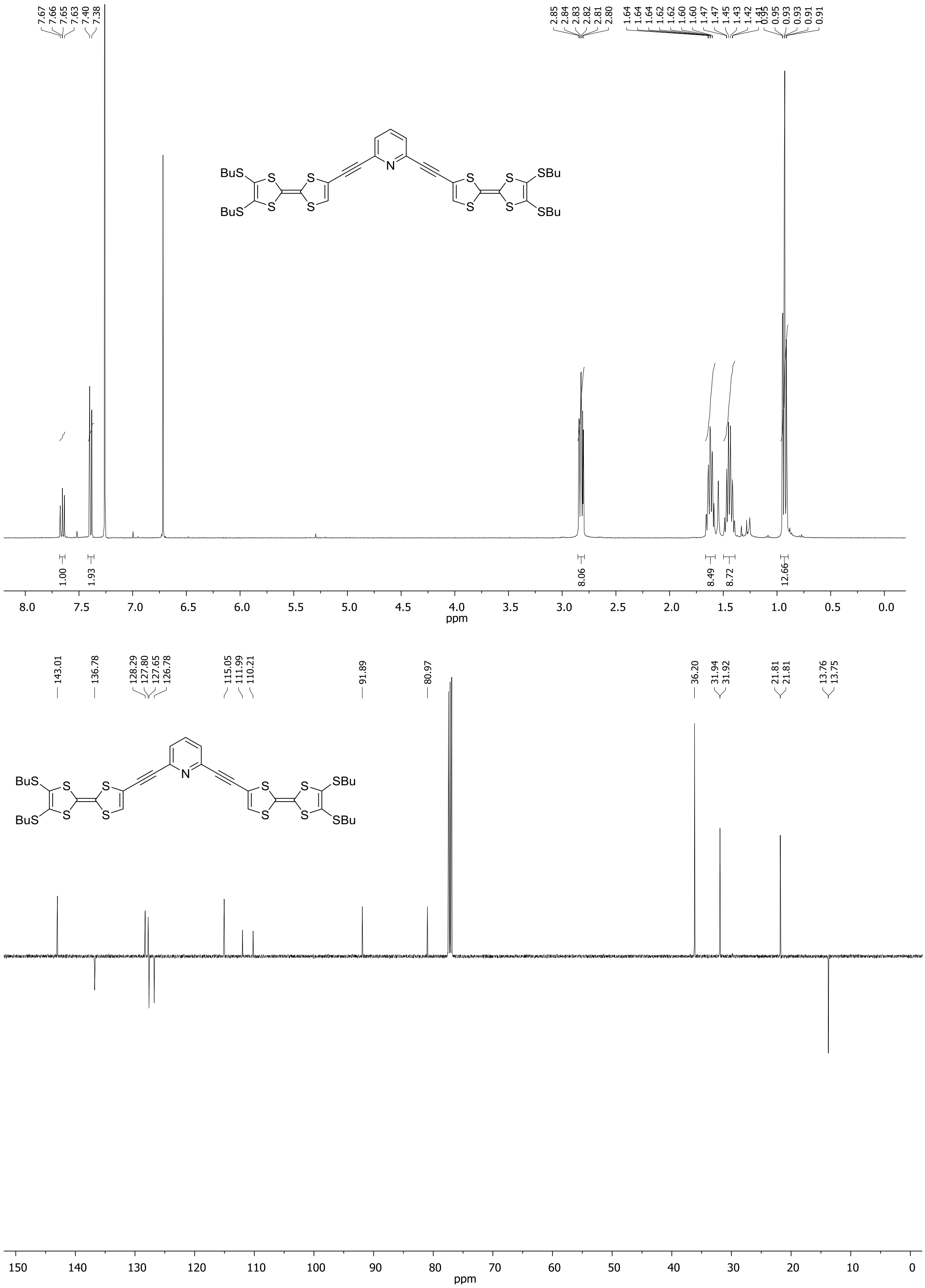

Figure S24: ^{1}H- and APT ^{13}C-NMR spectra of compound **7**.

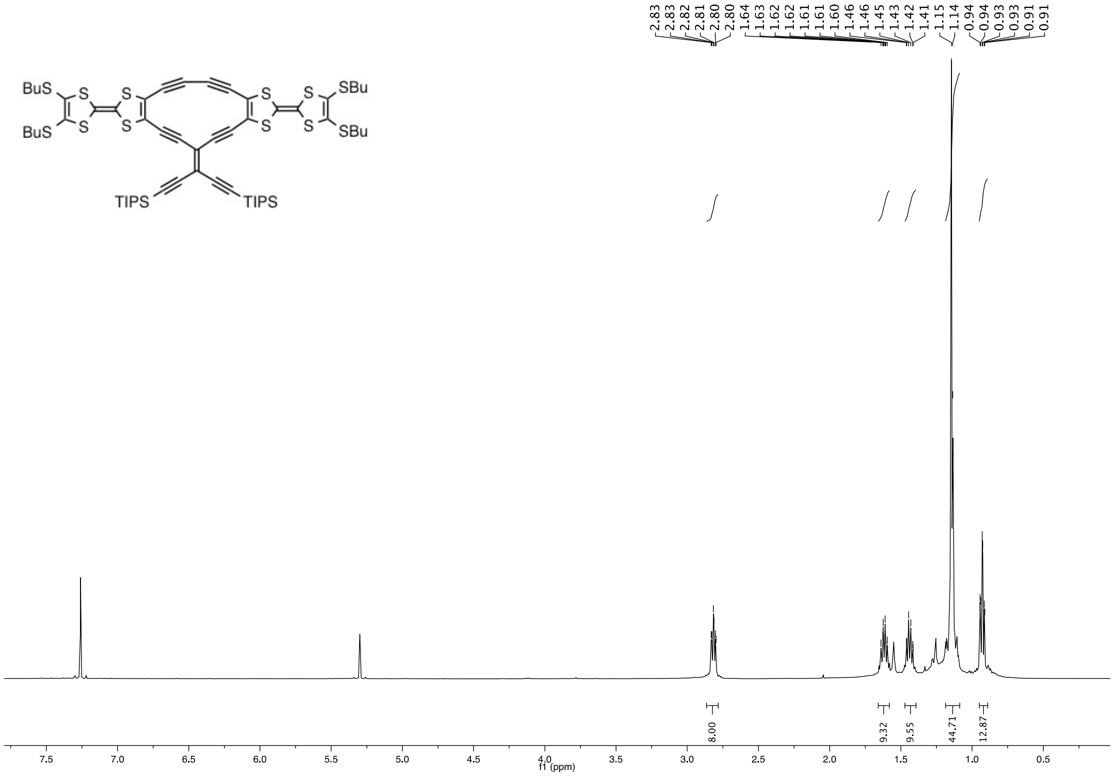

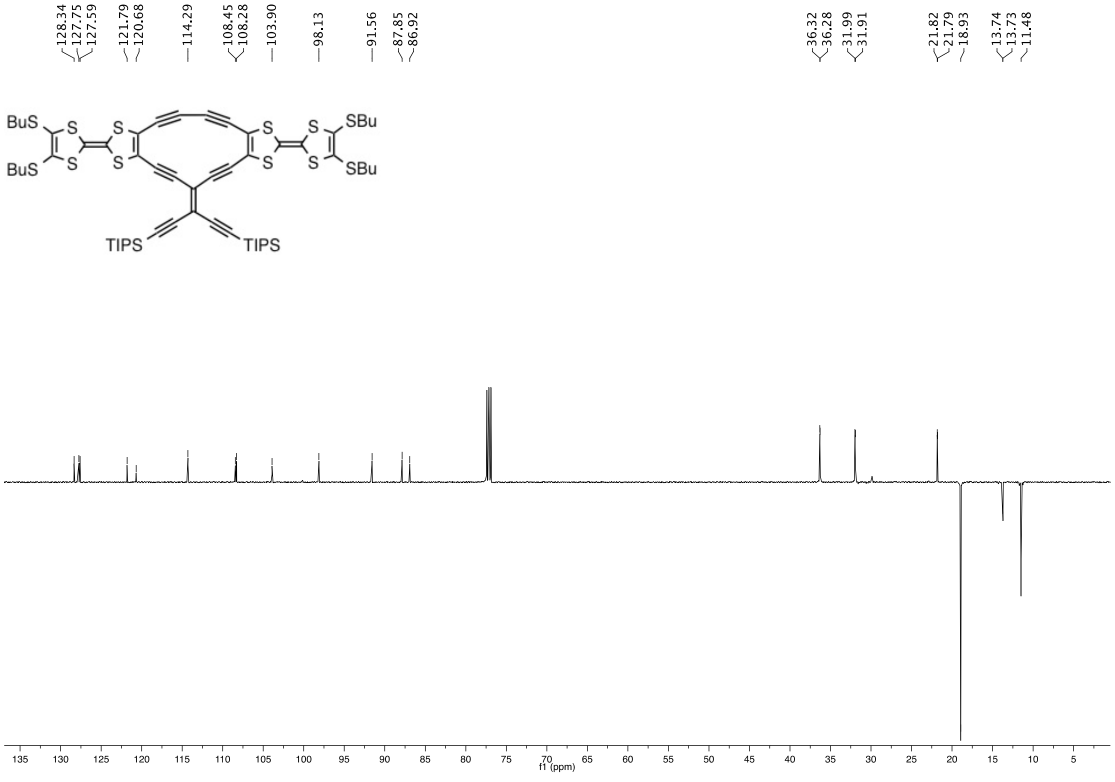

Figure S25: ^{1}H- and APT ^{13}C-NMR spectra of compound **8**.

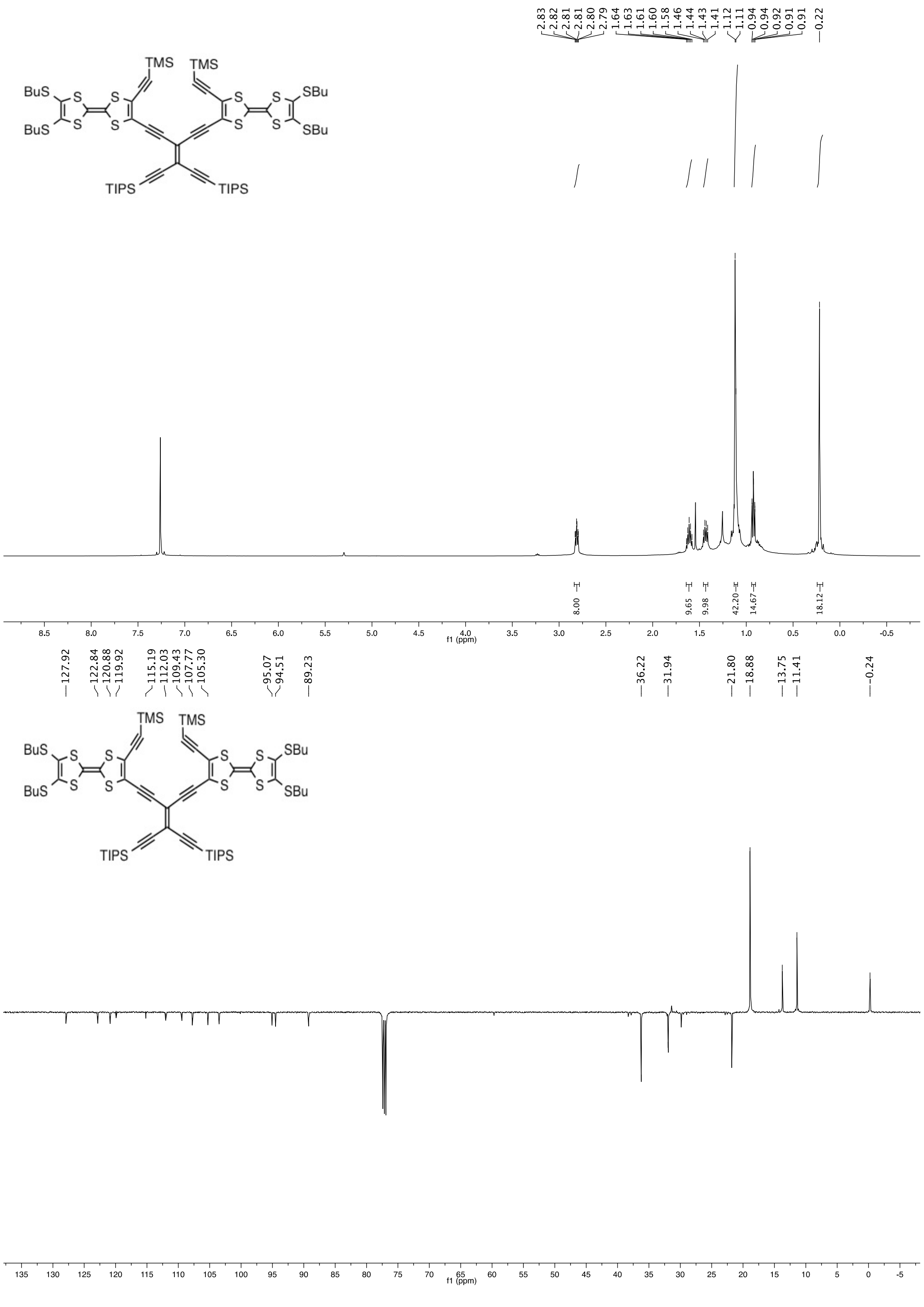

Figure S26: ^{1}H- and APT ^{13}C-NMR spectra of compound **14**.

IR spectrum of 8

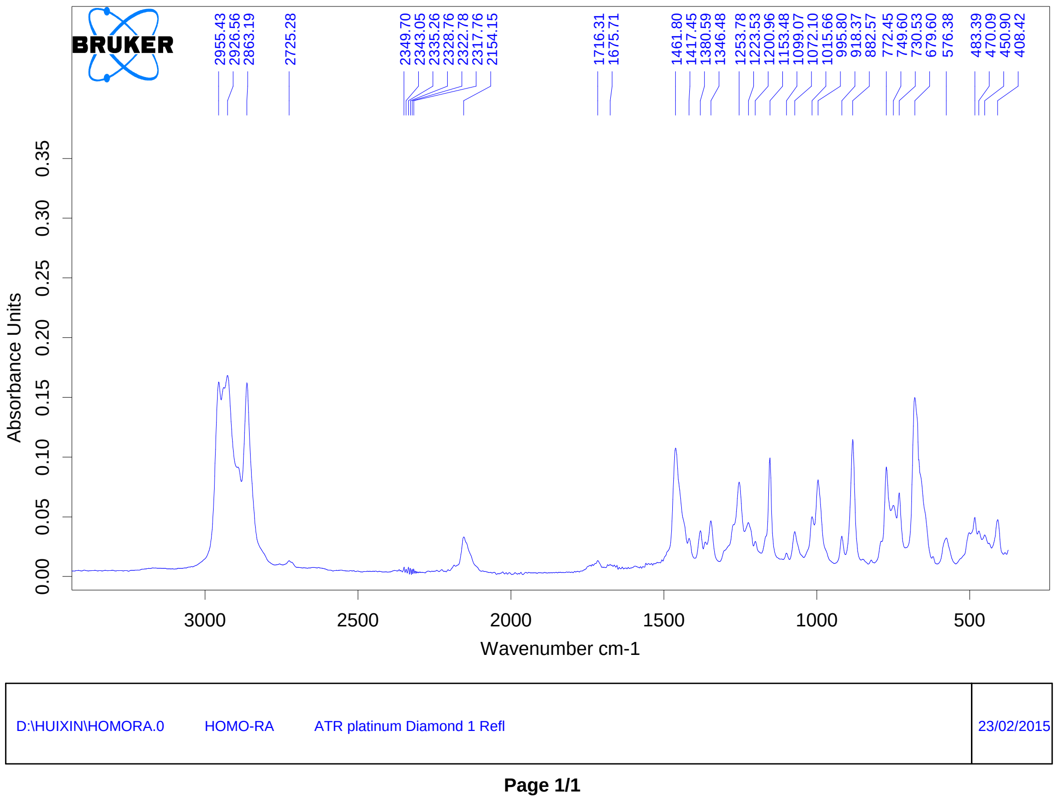

Figure S27: IR spectrum of compound **8**.

Calculations

Using **4** as a model compound, the interaction between the two neutral TTF units was studied by DFT calculations (RB3LYP cc-pVDZ). As seen in Figure S28 the energy barrier to be overcome in passing from **4**-*anti* to **4**-*syn* (SEt) by rotation amounts to only 2.5 kJ mol^{-1}. This is less than, for example, that observed in the classical case of ethane rotation [Quijano-Quiñones, R. F.; Quesadas-Rojas, M.; Cuevas, G.; Mena-Rejón, G. J. *Molecules* **2012**, *17*, 4661-4671, and references cited therein] and shows that the two TTF units in **4** can rotate essentially freely around the connecting -C≡C- bond system.

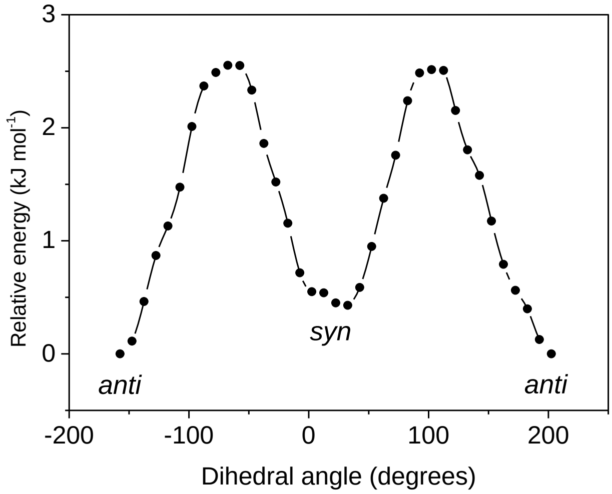

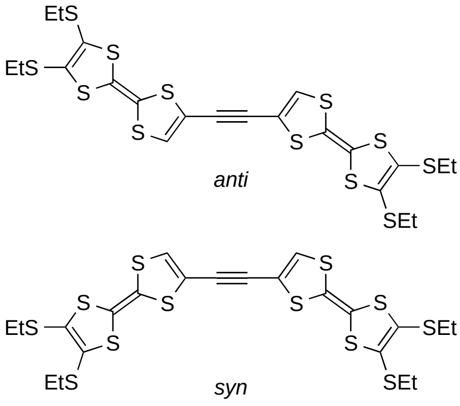

Figure S28: Energy profile for scanning the dihedral angle between the two TTF units in **4** (SEt) from *anti* to *syn*.

Density Functional Theory calculations (B3LYP/cc-pVDZ) were performed using *Gaussian 09*:

Gaussian 09, EM64L-G09RevB.01, Frisch, M. J.; Trucks, G. W.; Schlegel, H. B.; Scuseria, G. E.; Robb, M. A.; Cheeseman, J. R.; Scalmani, G.; Barone, V.; Mennucci, B.; Petersson, G. A.; Nakatsuji, H.; Caricato, M.; Li, X.; Hratchian, H. P.; Izmaylov, A. F.; Bloino, J.; Zheng, G.; Sonnenberg, J. L.; Hada, M.; Ehara, M.; Toyota, K.; Fukuda, R.; Hasegawa, J.; Ishida, M.; Nakajima, T.; Honda, Y.; Kitao, O.; Nakai, H.; Vreven, T.; Montgomery, Jr., J. A.; Peralta, J. E.; Ogliaro, F.; Bearpark, M.; Heyd, J. J.; Brothers, E.; Kudin, K. N.; Staroverov, V. N.; Keith, T.; Kobayashi, R.; Normand, J.; Raghavachari, K.; Rendell, A.; Burant, J. C.; Iyengar, S. S.; Tomasi, J.; Cossi, M.; Rega, N.; Millam, J. M.; Klene, M.; Knox, J. E.; Cross, J. B.; Bakken, V.; Adamo, C.; Jaramillo, J.; Gomperts, R.; Stratmann, R. E.; Yazyev, O.; Austin, A. J.; Cammi, R.; Pomelli, C.; Ochterski, J. W.; Martin, R. L.; Morokuma, K.; Zakrzewski, V. G.; Voth, G. A.; Salvador, P.; Dannenberg, J. J.; Dapprich, S.; Daniels, A. D.; Farkas, O.; Foresman, J. B.; Ortiz, J. V.; Cioslowski, J.; Fox, D. J. Gaussian, Inc., Wallingford CT, **2010**.

Structure optimizations were carried out to the opt=tight level and the lack of negative frequencies in the frequency calculations was taken as an indication that true minima had indeed been obtained.

DFT input for dihedral scan of 4 (SEt), Figure S27.

```
# opt=modredundant rb3lyp/cc-pvdz geom=connectivity
 -------------------------------------------------
 1/14=-1,18=120,19=15,26=3,38=1,57=2/1,3;
 2/9=110,12=2,17=6,18=5,40=1/2;
 3/5=16,11=2,16=1,25=1,30=1,71=1,74=-5,116=1/1,2,3;
 4//1;
 5/5=2,38=5/2;
 6/7=2,8=2,9=2,10=2,28=1/1;
 7//1,2,3,16;
 1/14=-1,18=20,19=15/3(2);
 2/9=110/2;
 99//99;
 2/9=110/2;
 3/5=16,11=2,16=1,25=1,30=1,71=1,74=-5,116=1/1,2,3;
 4/5=5,16=3/1;
 5/5=2,38=5/2;
 7//1,2,3,16;
 1/14=-1,18=20,19=15/3(-5);
 2/9=110/2;
 6/7=2,8=2,9=2,10=2,19=2,28=1/1;
 99/9=1/99;
 ----------------------------------------------------------
 TTF-CC-TTF-SEt n0 - scan the angle between the two TTF units
 ----------------------------------------------------------
 Symbolic Z-matrix:
 Charge =  0 Multiplicity = 1
 C                    -0.55876  -0.24918  -0.88594
 C                     0.55876   0.24916  -0.88595
 C                     1.86217   0.78554  -0.89422
 C                     2.18521   2.09248  -0.73159
 S                     3.23496  -0.33343  -1.20564
 H                     1.44787   2.87885  -0.57243
 C                    -1.86217  -0.78556  -0.89422
 C                    -2.18521  -2.0925   -0.73158
 S                    -3.23496   0.33341  -1.20564
 H                    -1.44788  -2.87886  -0.57242
 S                     3.8699    2.56633  -0.82654
 C                     4.49069   0.88691  -0.85627
 C                     5.79218   0.57923  -0.64996
 S                     6.43147  -1.08605  -0.69888
 S                     7.06277   1.79306  -0.32659
 C                     7.94272  -0.69587   0.17923
 C                     8.23618   0.62027   0.33379
 S                    -3.86991  -2.56634  -0.82653
 C                    -4.49069  -0.88692  -0.85627
 C                    -5.79218  -0.57923  -0.64996
 S                    -6.43147   1.08605  -0.69889
 S                    -7.06277  -1.79306  -0.32659
 C                    -7.94272   0.69588   0.17922
 C                    -8.23619  -0.62026   0.33379
```

S	8.97828	-2.05473	0.64674
S	9.66339	1.32577	1.12201
S	-9.66339	-1.32576	1.12201
S	-8.97827	2.05474	0.64674
C	8.04619	-2.72091	2.10947
H	8.05631	-1.94427	2.88859
H	7.00608	-2.89397	1.79593
C	10.99866	0.95606	-0.11455
H	11.13049	-0.1351	-0.15244
H	10.65151	1.31057	-1.09633
C	-10.99866	-0.95604	-0.11454
H	-11.13049	0.13511	-0.15244
H	-10.65151	-1.31056	-1.09633
C	-8.04618	2.72092	2.10946
H	-8.0563	1.94427	2.88859
H	-7.00607	2.89397	1.79593
C	-12.28327	-1.65833	0.31245
H	-12.15299	-2.75147	0.35017
H	-12.61804	-1.31748	1.30548
C	-8.70912	4.00739	2.58936
H	-9.75591	3.8357	2.88756
H	-8.69727	4.78301	1.8072
C	12.28327	1.65835	0.31244
H	12.15298	2.75149	0.35016
H	12.61804	1.31751	1.30547
C	8.70913	-4.00738	2.58936
H	9.75592	-3.83569	2.88757
H	8.69728	-4.783	1.80721
H	13.08641	1.43479	-0.4084
H	8.16921	-4.39949	3.4664
H	-8.16919	4.3995	3.46639
H	-13.08642	-1.43477	-0.40839

The following ModRedundant input section has been read:
D 5 3 7 9 S 36 10.000

DFT final output for dihedral scan of 4 (SEt), Figure13.

1\1\GINC-SLEJPNER\Scan\RB3LYP\CC-pVDZ\C22H22S12\HAMMERICH\25-Jan-2015\ 0\\# opt=modredundant rb3lyp/cc-pvdz geom=connectivity\\TTF-CC-TTF-SEt
n0 - scan the angle between the two TTF units\\0,1\C,-0.562856371,0.2
406098493,1.7696797314\C,0.562637015,-0.2394464484,1.7697264335\C,1.87
45783491,-0.7546010159,1.7780318166\C,2.2187477902,-2.0561664928,1.615
5945442\S,3.2290519609,0.3865151866,2.0892265771\H,1.4942437092,-2.854
3956979,1.4565818114\C,-1.8748223229,0.7556974949,1.7779282622\C,-2.21
91275752,2.0571754289,1.6150902261\S,-3.2291873749,-0.3854406784,2.089
5814531\H,-1.4947116035,2.8554241896,1.4557711135\S,3.9109063835,-2.50
26294102,1.7105526259\C,4.5043948092,-0.8133746741,1.7400466606\C,5.80
07299576,-0.4846750656,1.5337116192\S,6.4129817408,1.1907386002,1.5824
41712\S,7.0908119777,-1.6778313337,1.2105165917\C,7.9303983836,0.82496

43725,0.70445859\C,8.2451206389,-0.4862734412,0.5500625953\S,-3.911325
6146,2.5035092486,1.7099955114\C,-4.5046472076,0.8142051518,1.74001894
4\C,-5.8009448287,0.4852995173,1.5337756617\S,-6.4129891195,-1.1901821
077,1.582999371\S,-7.0911754187,1.6781859573,1.2102092963\C,-7.9303844
619,-0.824861846,0.7048033026\C,-8.2452915751,0.4862808147,0.550008282
\S,8.9438865548,2.2003310008,0.2368772209\S,9.6836071655,-1.1686664129
,-0.2380031292\S,-9.6838229455,1.1682486177,-0.2383333168\S,-8.9436175
036,-2.2005356137,0.2375239833\C,8.0011859071,2.8513350691,-1.22588823
5\H,8.0237705953,2.0748896477,-2.0049473307\H,6.9584291118,3.007681545
9,-0.9123465147\C,11.0126364066,-0.7773168631,0.998622556\H,11.1268768
198,0.3158284677,1.0363575067\H,10.671128601,-1.1372154507,1.980429298
3\C,-11.0128848807,0.77697264,0.9982550392\H,-11.1269199299,-0.3161842
173,1.0362881581\H,-10.6715725837,1.1372132705,1.9800050468\C,-8.00073
29957,-2.8517023244,-1.2250293409\H,-8.0235444359,-2.0755153557,-2.004
341656\H,-6.95792508,-3.0076298843,-0.9114450304\C,-12.3087692366,1.45
81738498,0.5711753104\H,-12.1963180012,2.5532911222,0.5333263288\H,-12
.6379377855,1.1118046675,-0.4218100949\C,-8.6424875032,-4.1489721449,-
1.7046458455\H,-9.6919402135,-3.994527403,-2.0029275937\H,-8.617959534
2,-4.9241123148,-0.9223061791\C,12.3084202236,-1.4589024636,0.57186998
25\H,12.1957471313,-2.5540064469,0.5343174855\H,12.6377651303,-1.11288
01352,-0.4211785285\C,8.6433654721,4.1482507948,-1.7058808978\H,9.6927
79838,3.993377845,-2.0040754452\H,8.619057138,4.9236398758,-0.92378101
54\H,13.1077968109,-1.2223276106,1.2927499601\H,8.0972118134,4.5315745
823,-2.5829409421\H,-8.0961727911,-4.5323797388,-2.5815678739\H,-13.10
81685411,1.2216417832,1.2920433606\\Version=EM64L-G09RevB.01\State=1-A
\HF=-5629.9617918,-5629.9617493,-5629.9616158,-5629.9614606,-5629.9613
612,-5629.9612305,-5629.9610261,-5629.9608894,-5629.9608439,-5629.9608
196,-5629.9608202,-5629.9609034,-5629.9610831,-5629.9612129,-5629.9613
517,-5629.961519,-5629.9615824,-5629.9615864,-5629.9616204,-5629.96162
85,-5629.9615682,-5629.9614303,-5629.9612678,-5629.9611229,-5629.96093
9,-5629.9608455,-5629.9608341,-5629.9608371,-5629.9609721,-5629.961104
7,-5629.9611906,-5629.9613445,-5629.9614904,-5629.961578,-5629.9616406
,-5629.9617436,-5629.9617918\RMSD=5.440e-09,3.521e-09,5.425e-09,6.316e
-09,9.844e-09,8.928e-09,7.208e-09,6.857e-09,3.611e-09,3.434e-09,7.777e
-09,5.681e-09,5.111e-09,4.340e-09,4.500e-09,8.796e-09,3.906e-09,5.052e
-09,8.201e-09,6.674e-09,7.477e-09,9.191e-09,5.650e-09,3.273e-09,3.475e
-09,8.556e-09,5.308e-09,9.360e-09,4.222e-09,3.748e-09,7.635e-09,6.456e
-09,7.511e-09,6.929e-09,6.171e-09,6.582e-09,6.818e-09\PG=C01 [X(C22H22
S12)]\\@

Intermolecular distance in hypothetical dimer structure

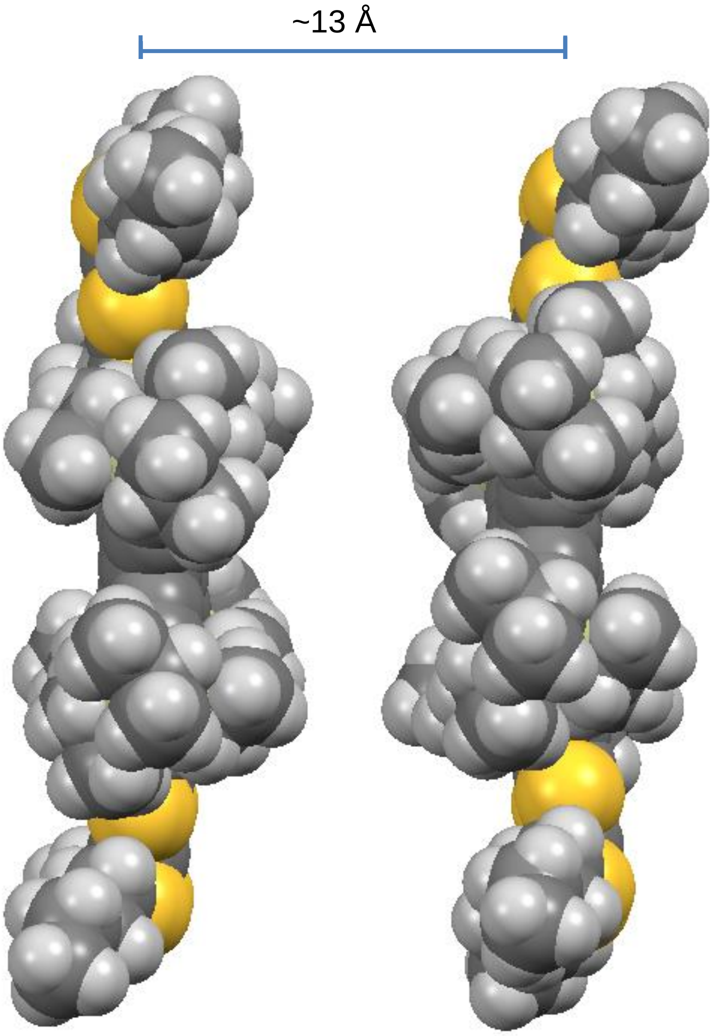

Figure S29: Estimated distance in dimer [(**2b**)$_2$]$^+$ (based on DFT calculations, UB3LYP/STO-3G).
